# Fitness, behavioral, and energetic trade‐offs of different migratory strategies in a partially migratory species

**DOI:** 10.1002/ecy.4151

**Published:** 2023-09-07

**Authors:** Andrea Soriano‐Redondo, Aldina M. A. Franco, Marta Acácio, Ana Payo‐Payo, Bruno Herlander Martins, Francisco Moreira, Inês Catry

**Affiliations:** ^1^ Helsinki Lab of Interdisciplinary Conservation Science (HELICS), Department of Geosciences and Geography University of Helsinki Helsinki Finland; ^2^ CIBIO, Centro de Investigação em Biodiversidade e Recursos Genéticos, InBIO Laboratório Associado, Campus de Vairão Universidade do Porto Vairão Portugal; ^3^ CIBIO, Centro de Investigação em Biodiversidade e Recursos Genéticos, InBIO Laboratório Associado, Instituto Superior de Agronomia Universidade de Lisboa Lisbon Portugal; ^4^ BIOPOLIS Program in Genomics Biodiversity and Land Planning, CIBIO, Campus de Vairão Vairão Portugal; ^5^ School of Environmental Sciences University of East Anglia Norwich UK; ^6^ School of Zoology, Faculty of Life Sciences Tel Aviv University Tel Aviv Israel; ^7^ School of Biological Sciences University of Aberdeen Aberdeen UK; ^8^ REN Biodiversity Chair, CIBIO/InBIO‐UP, Centro de Investigação em Biodiversidade e Recursos Genéticos Universidade do Porto, Campus Agrário de Vairão Vairão Portugal

**Keywords:** breeding success, GPS tracking, movement, overall dynamic body acceleration, survival

## Abstract

Alternative migratory strategies can coexist within animal populations and species. Anthropogenic impacts can shift the fitness balance between these strategies leading to changes in migratory behaviors. Yet some of the mechanisms that drive such changes remain poorly understood. Here we investigate the phenotypic differences, and the energetic, behavioral, and fitness trade‐offs associated with four different movement strategies (long‐distance and short‐distance migration, and regional and local residency) in a population of white storks (*Ciconia ciconia*) that has shifted its migratory behavior over the last decades, from fully long‐distance migration toward year‐round residency. To do this, we tracked 75 adult storks fitted with GPS/GSM loggers with tri‐axial acceleration sensors over 5 years, and estimated individual displacement, behavior, and overall dynamic body acceleration, a proxy for activity‐related energy expenditure. Additionally, we monitored nesting colonies to assess individual survival and breeding success. We found that long‐distance migrants traveled thousands of kilometers more throughout the year, spent more energy, and >10% less time resting compared with short‐distance migrants and residents. Long‐distance migrants also spent on average more energy per unit of time while foraging, and less energy per unit of time while soaring. Migratory individuals also occupied their nests later than resident ones, later occupation led to later laying dates and a lower number of fledglings. However, we did not find significant differences in survival probability. Finally, we found phenotypic differences in the migratory probability, as smaller sized individuals were more likely to migrate, and they might be incurring higher energetic and fitness costs than larger ones. Our results shed light on the shifting migratory strategies in a partially migratory population and highlight the nuances of anthropogenic impacts on species behavior, fitness, and evolutionary dynamics.

## INTRODUCTION

The migratory strategies of many animal species are rapidly changing due to anthropogenic influences, such as land transformation and climate change (Cox, 2010; Maclean et al., 2008; Sutherland, 1998; Visser et al., 2009). These changes are multifaceted and can encompass modifications in the timing of migration departure and arrival (Cotton, 2003; Gordo & Sanz, 2006; Jenni & Kéry, 2003), the shortening or diversion of migratory routes (Eichhorn et al., 2009; Sutherland, 1998), or the complete disruption of migration and the transition toward residency (Plummer et al., 2015; Pulido & Berthold, 2010; Satterfield et al., 2015). Ultimately, these adjustments can influence ecological and evolutionary processes at multiple scales, from the individual to the ecosystem (Dingle, 2014; Nathan et al., 2008).

Substantial within‐population variability can exist in the propensity to migrate, with some individuals from the population being resident and others migrating (Chambon et al., 2018; Lok et al., 2017; Sanz‐Aguilar et al., 2012). In many species and populations, migration strategies form a continuum and many alternative strategies coexist, such as long‐, medium‐, or short‐distance migrations, ranging movements, or localized residency, which generates partially migratory populations (Reid et al., 2018). Several studies have shown that migration is energetically costly, with individuals traveling longer distances incurring higher energy costs, often measured using the overall dynamic body acceleration (ODBA) (Flack et al., 2016; Somveille et al., 2018). Additionally, migration has been shown to increase mortality in a diverse range of taxa (Buchan et al., 2020; Klaassen et al., 2016; Rotics et al., 2017; Sillett & Holmes, 2002, but see Conklin et al., 2017). Yet, in some cases, selective pressures on survival fluctuate, with migrants presenting lower survival rates in years with average climatic conditions, but higher survival in years with extreme weather events (Acker, Burthe, et al., 2021; Acker, Daunt, et al., 2021; Sanz‐Aguilar et al., 2012).

The migratory strategy might also affect breeding performance. For instance, in European shags (*Gulosus aristotelis*) and older Eurasian spoonbills (*Platalea leucorodia*) individuals performing longer migrations breed later than short‐distance migrants or resident individuals, and late breeders have lower reproductive outcomes (Grist et al., 2017; Lok et al., 2017). Therefore, innovations in the migration strategy can be under strong selection if they provide individual fitness advantages over the rest, and can be retained and spread across the population through social learning or evolutionary change (de Zoeten & Pulido, 2020; Newton, 2008). In extreme cases, partial migration can become an unstable strategy and migration could even disappear if migratory individuals suffer increased fitness‐associated costs.

The advantages for an individual of adopting either a migratory or a resident strategy can be dependent upon its phenotype (Chapman et al., 2011). For example, in house finches (*Carpodacus mexicanus*), small individuals that cannot endure extremely cold temperatures, or large individuals less able to tolerate heat, migrate to areas with milder climates (Able & Belthoff, 1998; Belthoff & Gauthreaux Jr., 1991). Density‐dependent intraspecific competition can also play a role in maintaining partial migration (Chapman et al., 2011; Lundberg, 1987, 2013); in blue tits (*Cyanistes caeruleus*) and blackbirds (*Turdus merula*) smaller subordinate individuals migrate to avoid competition for limited resources, while larger dominant individuals remain in the breeding grounds year‐round (Lundberg, 1985; Nilsson et al., 2008; Smith & Nilsson, 1987). This leads to a frequency‐dependent evolutionary stable state and can induce highly dynamic temporal patterns on the ratio of resident to migratory individuals in the population (Chapman et al., 2011). Differences in the trophic niche specialization among individuals can also explain differential migration strategies, with individuals whose trophic niche is more affected by seasonal changes being more prone to migrate (Aparicio, 2000). In such cases, partial migration results in an evolutionarily stable strategy, where the fitness consequences for individuals that migrate are balanced against the consequences of remaining in the breeding area throughout the year (Buchan et al., 2020; Chapman et al., 2011).

Additionally, human activities can alter the trade‐offs between migratory strategies by providing a competitive advantage or disadvantage to individuals following a certain strategy (Buchan et al., 2020). However, the mechanisms that tip the balance between strategies remain largely unknown. The white stork (*Ciconia ciconia*) provides a unique opportunity to study the factors favoring the emergence of residency, as it displays a range of migratory strategies with different effects in terms of behavior, energetics, and fitness. For example, a comparison across eight white stork populations following diverse migratory strategies revealed that energy expenditure invested in locomotion increased with distance traveled (Flack et al., 2016), while a study on juvenile white storks found that individuals that migrated to Africa presented a lower survival, and increased movements, foraging range, and energy than those overwintering in Europe (Rotics et al., 2017).

Here, we assess the phenotypic differences, trade‐offs, and fitness consequences of migration in adult white storks from a partially migratory population breeding in Portugal that is transitioning toward residency (Catry et al., 2017; Gilbert et al., 2016). Whereas juvenile white storks perform annual migrations to Africa during their first year of life, when they reach adulthood, individuals show a range of fixed seasonal migratory strategies (Acácio et al., 2022; Catry et al., 2017; Marcelino et al., 2023). Some individuals are year‐round residents, remaining either locally or regionally in Southwest Europe; others migrate during the wintering period to Northwest Africa or the sub‐Sahara region (Catry et al., 2017). Markedly, the number of white storks breeding in Portugal has increased substantially in the last two decades, from approximately 3300 individuals in 1994 to 11,700 in 2017 (Catry et al., 2017). Simultaneously, the percentage of resident individuals has steeply increased, from 18% in 1995 to 62% in 2015 (Catry et al., 2017). This shift toward residency is likely to have been due to increased food availability (Catry et al., 2017) and milder temperatures during the winter in the breeding grounds. Landfill waste has become a key food resource for white storks, with individuals attending landfill sites on 44% of the days during the breeding season, and 60% of the days during the wintering season (Soriano‐Redondo et al., 2021).

Current trends suggest that partial migration in the Portuguese white stork population is not an evolutionarily stable strategy, as residents are disproportionally increasing in numbers (Catry et al., 2017). Thus, this provides a rare opportunity to investigate the ecological and evolutionary consequences of different migratory strategies throughout the annual cycle. Specifically, we investigated (1) the behavioral differences and estimated energetic costs, measured through ODBA, of birds undertaking various migratory strategies; (2) the fitness consequences of migration in terms of survival and reproduction; and (3) whether individual phenotype affects the migratory probability of adult white storks. We predict that migratory individuals will present higher energy expenditure because most evidence suggests that migrating is energetically costly (Flack et al., 2016). We also predict that migrants will suffer higher fitness costs (Buchan et al., 2020; Rotics et al., 2017), as is reflected by the current population shift toward residency.

## MATERIALS AND METHODS

### Fieldwork

We captured, measured, blood‐sampled, ringed, and tagged 75 breeding adult white storks in Southern Portugal between 2016 and 2020 (4 in 2016, 13 in 2017, 8 in 2018, 43 in 2019, and 7 in 2020). Storks were captured either at their nest using remotely activated clap nets or at landfill sites using nylon leg nooses. GPS/GSM loggers (Movetech Telemetry and Ornitela) were mounted on the backs of the birds as backpacks with a Teflon harness; the total weight of the logger and harness was 60–90 g, 1.5%–3.7% of the bird body mass. The loggers were programmed to record GPS positions and tri‐axial acceleration samples every 20 min at 1 Hz for 9 s. At deployment, morphometric measurements (wing, tarsus, and bill length ± 1 mm and weight ± 1 g) were taken for each individual. Blood (<50 μL) was collected from the medial metatarsal vein and a few drops were preserved in vials with ethanol for molecular sexing. All birds were colored‐ringed following a unique scheme. All procedures were performed under license of the Instituto da Conservação da Natureza e Florestas, Portugal (license numbers: 493/2016/CAPT, 661/2017/CAPT, 662/2017/CAPT, 548/2018/CAPT, 549/2018/CAPT, 248/2019/CAPT, 365/2020/CAPT, 366/2020/CAPT, and 367/2020/CAPT). Approval from an ethics committee was not required for this study.

Nesting sites were identified for all adults by visually inspecting GPS tracks, and they were visited weekly during the breeding season in subsequent years to determine stork breeding parameters (i.e., laying date, and number of fledglings). Nests were visited annually during the breeding season after the logger stopped recording to assess if this was due to tag failure or bird mortality; this was a reliable method due to high levels of nest faithfulness. Moreover, when an individual was not found in the nest it had used in the previous year, other nests of the colony and nearby colonies were also visited to confirm if the individual had not moved to a neighboring nest. In total, ~420 nests were monitored on a weekly basis during the 2016–2020 breeding seasons.

### GPS and acceleration data

We used the 9 s tri‐axial acceleration bursts to calculate two movement parameters: ODBA (G), a proxy of energy expenditure invested in locomotion, and bird behavior (Gleiss et al., 2011; Shepard et al., 2008). Following Soriano‐Redondo et al. (2021), to calculate ODBA we subtracted the smoothing of the acceleration, using a running mean of 4 s, from the total acceleration. To estimate the bird behavior at each burst we trained two random forest machine‐learning algorithms, one for Movetech Telemetry tags and the other for Ornitela tags, using 1000 manually labeled tri‐axial acceleration bursts encompassing four behaviors: foraging, resting, soaring and flapping (for details see Soriano‐Redondo et al., 2021). The random forest models had 96% accuracy for Movetech Telemetry tags and 97% accuracy for Ornitela tags.

### Characterization of migration strategies

We used the GPS trajectories to classify the migratory strategy of each individual every year. We visually examined the GPS data to detect and remove potential outliers. Storks were classified as resident or migratory depending on whether they remained in Southwest Europe or crossed the Strait of Gibraltar after the breeding period. Birds were subsequently classified into four subcategories depending on their wintering grounds. Resident birds were categorized as either local when they remained in proximity to the nest year around (i.e., <50 km away from it); or regional, when they ranged further away from the nest across Southwest Europe (i.e., >50 km away from the nest). We chose this threshold as it ensured that birds classified as local did not commute between different areas in Portugal and always remained close to their nesting site. Migrants who crossed the Strait of Gibraltar and spent the winter in Northwest Africa were classified as Northwest African, and sub‐Saharan when they crossed the Sahara Desert as well and wintered in the Sahel.

To establish the migratory phenology of tracked birds we followed Soriano‐Redondo et al. (2020). Each annual cycle was divided into four seasons: autumn, wintering, spring, and breeding. For migratory individuals, we defined the start of autumn and spring (i.e., migrations) as the first day a bird moved >60 km between roosts for 3 days consecutively, which led to the departure of the breeding range during autumn migration, and the wintering range during spring migration. The end of autumn and spring was the last day the bird moved >60 km between roosts for 3 days consecutively, after departing from the wintering range during autumn migration, and from the breeding range during spring migration. For resident individuals, we derived the thresholds between periods using the median date of the thresholds of the migratory birds. The start of the autumn period was the 4 August and the end was on 5 September; the start of the wintering period began on 6 September and the end was on 12 December; the start of the spring period was on 13 December and the end was on 22 January; and the start of breeding period was on 23 January and the end was on 3 August.

### Breeding parameters estimation

The nest occupation date was determined using the GPS locations, and was defined as the first day that a bird visited its nest for 3 days consecutively. Laying date and number of fledglings were determined by regularly examining the nests using a camera attached to a pole, or by using a drone.

### Statistical analysis

We explored the potential effects of migratory strategy (four levels: local, regional, Northwest Africa, and sub‐Saharan) on bird movements and ODBA. To do that, we first fitted a linear mixed model (LMM) with annual displacement (i.e., the sum of all the distances moved throughout the year) as the response variable and migratory strategy as explanatory variables. To control for potential differences in tag recordings and individual behavior, we included the number of GPS positions as a fixed factor and individual IDs as a random effect. Second, to understand the implications of the different migratory strategies on the annual energy expenditure, we fitted a linear mixed effects model with mean annual ODBA as the response variable, migratory strategy as the explanatory variable, and individual ID nested in tag type (five levels: four tag types from Movetech Telemetry and one from Ornitela) as a random effect. We included tag type to account for different sensitivities of the tags to record the acceleration measures. Third, to understand the differences in ODBA linked to foraging, resting, soaring, and flapping among the four migratory strategies, we fitted four LMM with mean annual ODBA during foraging, resting, soaring, and flapping as response variables and migratory strategy as an explanatory variable, and individual ID nested in tag type as a random effect. To implement the models, we used the R package *lme4* (Bates et al., 2015, p. 4). To assess the differences between migratory strategies, whenever this variable was significant in the model, we performed Tukey's contrasts.

To understand the behavioral budgets associated with each migratory strategy we fitted generalized LMMs (GLMMs) with Beta distribution, using the R package *glmmTMB* (Brooks et al., 2017). The response variable was the mean proportion of time per day spent performing a certain behavior in an annual cycle. Thus, we fitted four models with the proportion of time foraging, resting, soaring, and flapping. In each, the explanatory variable was migratory strategy and individual ID nested in tag type was a random effect. In the cases in which migratory strategy was significant, we assessed the differences between groups by implementing Tukey's contrasts.

To understand at which stage of the seasonal cycle differences in the bird ODBA, movements, and behavior occurred, we fitted the same models as previously used, with migratory strategy and individual ID (nested in tag type for the ODBA and behavior parameters), but including season as well (four levels: autumn, wintering, spring, and breeding) and the interaction of migratory strategy and season as fixed effects. In this case, the response variables were seasonal displacement, mean seasonal ODBA, mean seasonal ODBA during foraging, resting, soaring, and flapping, and mean proportion of time per day spent foraging, resting, soaring, and flapping during the season. For seasonal displacement, because the duration of the season differed depending on the bird, we also included the duration as a covariate. We implemented Tukey's contrasts to assess the differences between seasons and migratory strategies.

**FIGURE 1:**
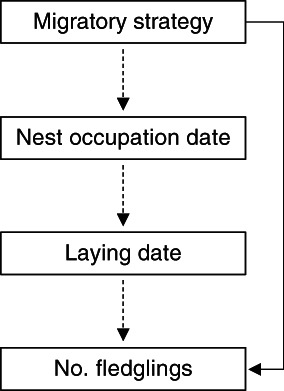
Path diagram of piecewise structural equation modeling to establish the direct (solid line) and indirect (dashed lines) relationship between the migratory strategy and the reproductive success.

We assessed the direct and indirect effects of the migratory strategies on the subsequent breeding performance. We tested whether migratory strategy directly affected the number of fledglings produced and/or whether there was a cascading effect, with migratory strategy affecting the number of fledglings through changes in nesting and laying dates, as has been reported in other species (Grist et al., 2017; Lok et al., 2017). To do so, we fitted a structural equation model containing three linear models (Figure 1) using the *piecewiseSEM* R package (Lefcheck, 2016; Lefcheck et al., 2016). We fitted an LMM with nest occupation date (day of the year) as a response variable, migratory strategy as the explanatory variable and individual ID as the random effect. This was followed by a LMM model linking laying date (as the response) and nest occupation date as a fixed effect, and with individual ID as the random effect. Last, we fitted a GLMM with a Poisson distribution with the number of fledglings as the response, laying date and migratory strategy as covariates, and individual ID as a random effect. The direct and indirect relationships were also tested outside the structural equation model to extract the effects.

Finally, to determine whether the probability of migration was linked to individual characteristics, we fitted a generalized linear model (GLM) with a binomial link function, with migration probability (resident or migrant) as the response variable, and wing length, sex, and their interaction as explanatory variables. Wing length is correlated with culmen (Pearson's correlation = 0.629, *p* < 0.001) and tarsus lengths (Pearson's correlation = 0.548, *p* < 0.001), and thus a good proxy of body size. Although males tend to be larger than females, collinearity between sex and wing size was relatively low (variance inflation factor [VIF = 1.83]). All model assumptions were checked using the *DHARMa* R package.

### Survival estimation

Survival, GPS signal loss and resighting probabilities were simultaneously estimated by means of multievent capture–recapture models (Pradel, 2005). The multievent framework distinguishes what can be observed in the field from the underlying biological states of the individuals, which must be inferred (Pradel, 2005). Live encounter data were collected during the breeding season of each year between 2016 and 2021 and coded into individual encounter histories. Here, the events were “0” for individuals not observed in a given year. Observed individuals were stratified according to whether they had an active GPS tag or not. We assigned “1” to individuals detected with active GPS devices and “2” to individuals observed alive that either had an inactive GPS device or had lost the GPS device but could be identified by means of rings. In addition to live encountered data, dead recoveries (*n* = 10) were detected by fixed location in GPS signal and confirmed by local scientists. Dead encounters were coded as “3.”

We specified the multievent model with three sets of parameters: (1) the initial state probabilities; (2) the state transition probabilities that included the probability of losing the GPS signal and the probability of survival; and (3) the probabilities of resight and recovery. The model included four underlying biological states: two states for live individuals, coded Aa (alive with active GPS) and Ai (alive with inactive GPS), and two states for dead individuals, coded Ra (recently dead with active GPS signal); and LD (long dead).

The multievent model (see details in Appendix S1: Section S3) estimated the probabilities of transition between the states (GPS signal loss and survival) and the probabilities of the events (resighting and recovery). Given our knowledge of the system, our starting model considered the following constraints: initial state probability (τ) was certain for every individual, as all individuals started as alive with an active GPS device deployed (τ_Aa = 1). Because all recoveries were from individuals with active GPS loggers, we fixed the recovery probability as 1 (*r* = 1). Likewise, the probability of resighting individuals with active GPS devices was fixed to 1 (*p*Aa = 1). Finally, the model included migratory strategy (resident or migratory) in resighting probability because the probability of resighting individuals without the GPS signal was higher for residents than for migrants. We ran two models, the first model to estimate survival as a function of the migratory strategy with four levels: local, regional, Northwest Africa and sub‐Saharan that could not estimate all the parameters due to the small sample size. Thus, we ran a second model, in which we only tested differences in survival between resident and migratory individuals. To test whether there were significant differences between migratory and resident individuals we compared Quasi‐Akaike Information Criterion (QAIC) values between this model and a null model, where only recapture probability was influenced by migratory strategy. We ran a goodness‐of‐fit test (GOF [Choquet et al., 2009]) in R2UCARE (Gimenez et al., 2018) that suggested the presence of transience effects, but this was not significant (χ^2^ = 9.3, *df* = 4, *p*‐value = 0.052). The remaining tests were not estimable. We used 2.52 as a VIF and used it to correct all models constructed in E‐SURGE (Choquet et al., 2005).

## RESULTS

**FIGURE 2:**
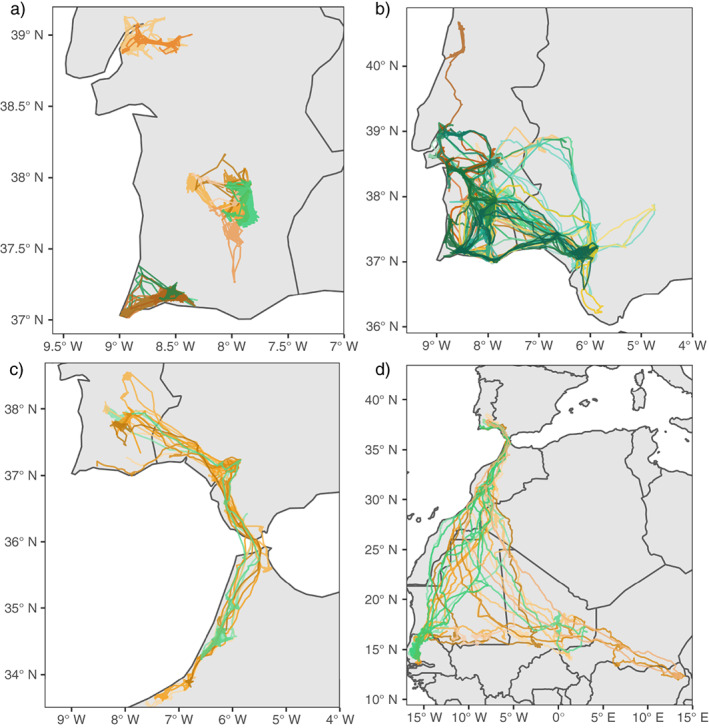
Migratory strategies of white storks breeding in Portugal that are resident (a) locally and (b) regionally, and those that migrate to (c) Northwest Africa and (d) sub‐Saharan Africa. Shades of green represent males and shades of orange females.

We tracked 75 adult white storks (36 males and 39 females) using GPS/GSM loggers equipped with acceleration sensors during a total of 212 annual cycles (78 complete annual cycles), from 2016 to 2021. Individuals displayed four different strategies: they remained in Southwest Europe either locally (Figure 2a) or regionally (Figure 2b), or they migrated and overwintered in Northwest Africa (Figure 2c) or sub‐Saharan Africa (Figure 2d). Overall, 58 individuals were residents spending the nonbreeding periods in Southwest Europe (9 locally and 42 regionally, and 7 changed across years), and 16 were migratory and spent the nonbreeding period in Africa (6 in Northwest Africa and 10 in sub‐Saharan Africa). With one exception, adult white storks tracked over multiple years were consistent in their tendency to migrate.

### Behavioral and energetic consequences of migration

**FIGURE 3:**
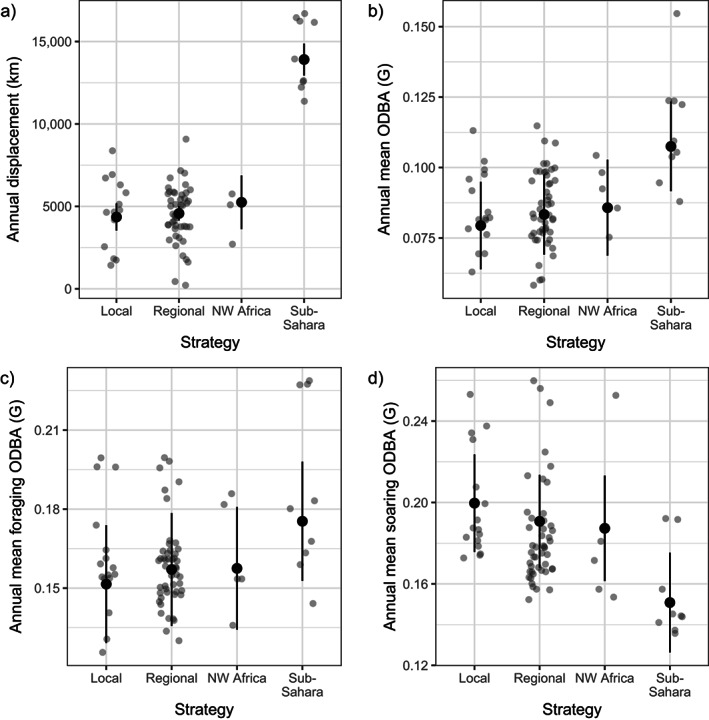
Relationship between the migratory strategy and (a) the annual displacement, (b) the annual mean overall dynamic body acceleration (ODBA) (G), and (c) the annual mean foraging ODBA (G). Black dots are predicted estimates from the linear mixed model, vertical lines are the 95% CIs based on fixed‐effect uncertainty, and gray dots are raw data. NW Africa, Northwest Africa.

Our results showed that the migratory strategy affected annual displacement (Figure 3a, migratory strategy: χ^2^ = 313.743, *p* < 0.001; number of GPS positions: χ^2^ = 44.931, *p* < 0.001), with sub‐Saharan winterers traveling thousands of kilometers more than all the other storks (Appendix S1: Table S1). A similar pattern was observed in annual ODBA derived from the acceleration sensors (Figure 3b, χ^2^ = 36.239, *p* < 0.001): individuals migrating to sub‐Saharan countries presented >20% higher ODBA than individuals that migrated to Northwest Africa or that remained in Southwest Europe (Appendix S1: Table S2). Finally, ODBA linked to foraging (Figure 3c, χ^2^ = 23.172, *p* < 0.001) and soaring (Figure 3d, χ^2^ = 70.927, *p* < 0.001) was also affected by the migratory strategy, but ODBA linked to resting and flapping was not (resting: χ^2^ = 1.806, *p* = 0.614; flapping: χ^2^ = 1.287, *p* = 0.732). Sub‐Saharan migrants presented *a* ~ 10% higher ODBA while foraging, and *a* ~ 25% lower ODBA while soaring than residents and birds that migrated to Northwest Africa (Appendix S1: Tables S3 and S4).

**FIGURE 4:**
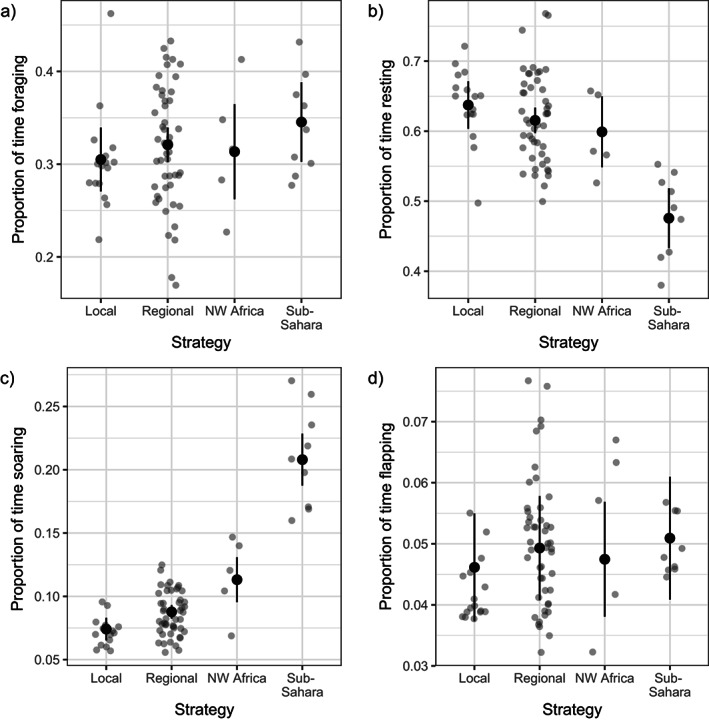
Relationship between white storks migratory strategy and the proportion of time (a) foraging, (b) resting, (c) soaring, and (d) flapping. Black dots are predicted estimates from the generalized linear mixed model, vertical lines are the 95% CIs based on fixed‐effect uncertainty, and gray dots are raw data. NW Africa, Northwest Africa.

The differences in ODBA could be partly mediated by differences in behavioral budgets. While the proportion of time devoted to foraging and flapping was similar across migratory strategies (Figure 4a; foraging: χ^2^ = 2.178, *p* = 0.536; Figure 4d, flapping: χ^2^ = 3.108, *p* = 0.375), birds that migrated to sub‐Saharan Africa spent overall less time resting (Figure 4b, χ^2^ = 40.2, *p* < 0.001; Appendix S1: Table S5) and more time soaring (Figure 4c, χ^2^ = 227.54, *p* < 0.001; Appendix S1: Table S6) compared with the remaining strategies.

Distance traveled varied depending on season (χ^2^ = 10.391, *p* = 0.015; Appendix S1: Figure S1) and migratory strategy (χ^2^ = 408.161, *p* < 0.001; Appendix S1: Figure S1), with a significant interaction between them (χ^2^ = 275.684, *p* < 0.001; Appendix S1: Figure S1). We controlled for the number of GPS positions (χ^2^ = 47.154, *p* < 0.001), and the duration of the season (χ^2^ = 14.258, *p* < 0.001). Sub‐Saharan migrants traveled longer distances during autumn, spring, and winter than birds that adopted other strategies (Appendix S1: Figure S1, Table S7). ODBA also differed among seasons (χ^2^ = 232.97, *p* < 0.001; Appendix S1: Figure S2) and migratory strategies (χ^2^ = 136.40, *p* < 0.001; Appendix S1: Figure S2), with a significant interaction effect (χ^2^ = 174.66, *p* < 0.001; Appendix S1: Figure S2). During both autumn and spring, migratory birds (including sub‐Saharan and Northwest Africa winterers) presented higher ODBA than birds that remained in Southwest Europe (both locally and regionally; Appendix S1: Figure S2, Table S8). During the winter, sub‐Saharan migrants continued to have higher ODBA than other birds, while during the breeding period all birds had similar levels of ODBA (Appendix S1: Figure S2, Table S8). We also found that ODBA during foraging and soaring varied depending on the season (foraging: χ^2^ = 190.665, *p* < 0.001; Appendix S1: Figure S3; soaring: χ^2^ = 26.219, *p* < 0.001; Appendix S1: Figure S4) and the migratory strategy (foraging: χ^2^ = 54.181, *p* < 0.001; Appendix S1: Figure S3; soaring: χ^2^ = 40.801, *p* < 0.001; Appendix S1: Figure S4), with a significant interaction effect (foraging: χ^2^ = 107.219, *p* < 0.001; Appendix S1: Figure S3; soaring: χ^2^ = 50.293, *p* < 0.001; Appendix S1: Figure S4). Interestingly, sub‐Saharan migrants had significantly higher ODBA while foraging during the autumn than resident birds, and during the winter in sub‐Saharan Africa compared with the other strategies. In the other seasons, however, all birds presented similar levels of ODBA (Appendix S1: Figure S3, Table S9). By contrast, sub‐Saharan migrants had significantly lower ODBA while soaring compared with residents and short‐distance migrants during both migrations and the wintering period (Appendix S1: Figure S4, Table S10).

Migratory strategy and season also affected the proportion of time devoted to each behavior (Appendix S1: Figures S5–S8, Tables S11–S15). Sub‐Saharan birds spent less time resting (Appendix S1: Figure S6, Table S16) and more time soaring (Appendix S1: Figure S7, Table S17) during both migrations and during winter. In addition, they allocated more time to foraging during the winter period and less during the spring (Appendix S1: Figure S5, Table S15). Birds that migrated to Northwest Africa, also increased soaring time and decreased resting time during both migrations, but their behavior during the winter period was similar to that of resident birds (Appendix S1: Figures S6 and S7, Tables S16–S18).

### Breeding success

**FIGURE 5:**
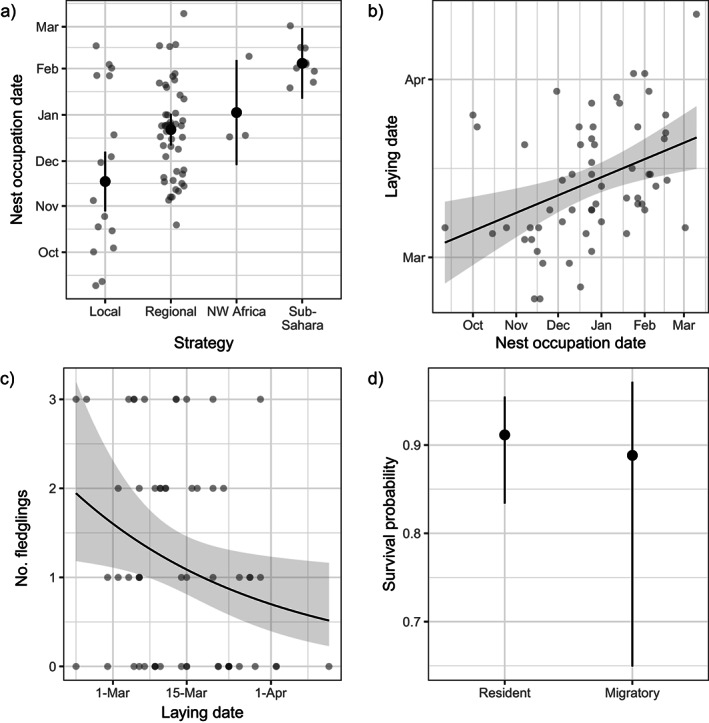
(a) Relationship between white storks migratory strategy and the nest occupation date. (b) Relationship between the nest occupation date and the laying date. (c) Relationship between the laying date and the number of fledglings. (d) Survival probability depending on whether the individual remains resident (locally or regionally) or migrates (to Northwest or sub‐Saharan Africa). Black dots and black lines are predicted estimates from the (generalized) linear mixed model and multievent capture–recapture model, vertical lines and gray shades are the 95% CIs based on fixed‐effect uncertainty, and gray dots are raw data. NW Africa, Northwest Africa.

We did not find a direct link between migratory strategy and number of fledglings raised, but we did find an indirect relationship between these two variables (Table 1). The migratory strategy of each individual affected its arrival time to the nest location (Figure 5a, χ^2^ = 25.697, *p* < 0.001): birds that moved across Southwest Europe occupied the nest significantly later than birds that remained locally (Tukey's contrasts: local–regional *z* = 3.092, *p* = 0.008), whereas sub‐Saharan migrants occupied their nest significantly later than resident birds (Tukey's contrasts: local–sub‐Saharan *z* = 5.021, *p* < 0.001; regional–sub‐Saharan *z* = 3.364, *p* = 0.004). In turn, a later occupancy of the nest led to a later laying date for those birds (Figure 5b; χ^2^ = 9.756, *p* = 0.002), which ultimately reduced breeding success, that is, birds laying eggs later raised a lower number of fledglings (Figure 5c; χ^2^ = 4.874, *p* = 0.027).

**TABLE 1:** Analysis of variance for the structural equation model to establish the direct and indirect relationship between the migratory strategy and the reproductive success.

Response	Predictor	*F*‐statistic	*df*	*p*
Nest occupation date	Migratory strategy	25.7	3	**<0.001**
Laying date	Nest occupation date	9.8	1	**0.0018**
No. fledglings	Laying date	5.1	1	**0.0239**
No. fledglings	Migratory strategy	5.1	1	0.0640

*Note*: Bold values represent *p* < 0.05.

### Survival

We did not find significant differences between residents (local and regional) and migrants (to Northwest and sub‐Saharan Africa) in survival probability, as the null model including only the effect of migration in recapture probability presented a lower QAIC_c_ than the full model (null model QAIC_c_ = 112.88; full model QAIC_c_ = 114.92; ΔQAIC_c_ = 2.05). However, the full model suggests that residents might be experiencing slightly higher survival than migrants (migrants: survival probability = 0.89, confidence interval [CI] = 0.65–0.97; residents: survival probability = 0.91, CI = 0.83–0.95; Figure 5d), but a larger sample size would be needed to confirm this. The probability of recapture when the signal had been lost was much lower for migrants (0.14) than residents (0.63).

### Phenotypic differences in migration strategy

**FIGURE 6:**
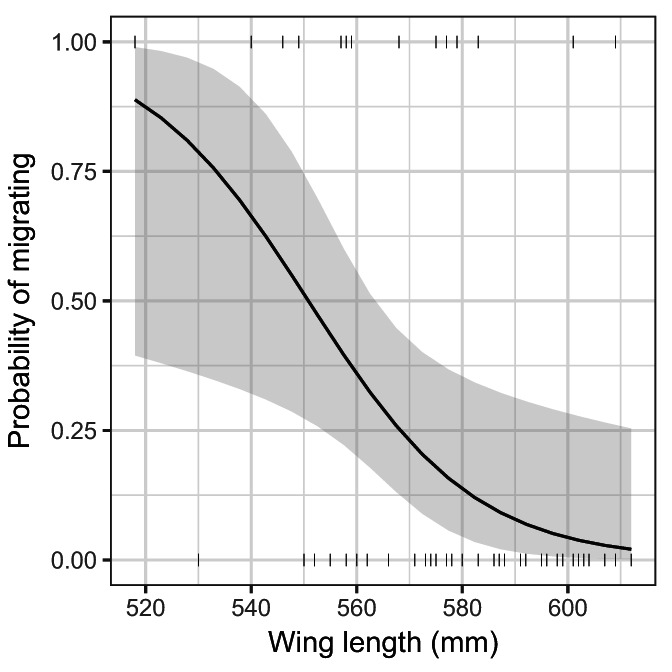
Relationship between white stork wing length (in millimeters) and the probability of migrating to Northwest Africa and sub‐Saharan Africa. Shading represents 95% CIs and vertical bars are raw data.

We found that migration probability was affected by wing length, a proxy for individual size (χ^2^ = 8.371, *p* = 0.004), but was not affected by sex (χ^2^ = 0.641, *p* = 0.423), or the interaction of wing length and sex (χ^2^ = 0.142, *p* = 0.706). The significant negative relationship between wing length and migration probability shows that larger birds were more likely to be resident while smaller birds tended to be migratory (Figure 6).

## DISCUSSION

In this study we showed the behavioral and energetic trade‐offs of different migratory strategies throughout the whole annual cycle of a long‐lived bird. While we do not find a direct effect of migration strategy on fitness, we did find that migratory birds occupied their nests later, and later occupation led to delayed laying dates and a lower number of fledglings. We also found that trans‐Saharan migrants traveled longer annual distances, spent more time flying and less resting, and incurred higher energetic costs than storks adopting other movement strategies. By contrast, individuals that migrated to Northwest Africa did not differ in behavior or energy expenditure from resident birds (except during spring and autumn). These results are in concordance with previous evidence from juvenile white storks that suggested that wintering in Europe was less demanding compared with sub‐Saharan Africa (Rotics et al., 2017).

The behavioral and energetic contrast between birds wintering in Southwest Europe or Northwest Africa and birds traveling to the Sahel is particularly acute during the winter, but also during spring and autumn, whereas during the breeding period all birds have a similar energy expenditure and behavior. These differences are likely to occur due to several factors. First, the Sahel is 2500 km away from the breeding grounds and reaching this wintering area requires substantial investments in terms of time and energy. Nevertheless, our results also showed that thermal conditions in the Sahel are likely to be more favorable, as reflected by the lower ODBA estimates of individuals while soaring (Flack et al., 2016). Previous evidence from juvenile white storks from Southwest Germany showed similar results, with individuals overwintering in Northwest Africa moving less during stopover days and having lower ODBA values compared with birds wintering south of the Sahara (Flack et al., 2016). By contrast, residents and individuals that overwintered in Northwest Africa had access to low‐cost foraging areas at landfills throughout the year (Ciach & Kruszyk, 2010; Flack et al., 2016; Marcelino et al., 2023), while sub‐Saharan migrants forage on natural prey in the Sahel (Elliott et al., 2020), which is likely to be energetically more expensive, as reflected by their higher foraging ODBA. Finally, the longer daylight availability in the Sahel region, compared with Southwest Europe and Northwest Africa during the nonbreeding period, could enable sub‐Saharan individuals to increase their diurnal movement activities (Pokrovsky et al., 2021).

Our results show that trans‐Saharan migrants present higher ODBA, a proxy for energy expenditure, than storks adopting other movement strategies, but we could not quantify the absolute or relative differences in energy expenditure between migratory strategies, as we could not calibrate the relationship between ODBA and energy expenditure (Halsey & Bryce, 2021). Data on the daily energy expenditure of juvenile white storks (quantified using continuous heart rate and fine‐scale movement tracking of the individuals) show that their heart rate increases linearly with ODBA and supports our conclusions (Flack et al., 2020). However, other physiological factors may also influence energy expenditure, the costs for thermoregulation and hydroregulation can be significantly different for individuals overwintering in Southwest Europe and Northwest Africa compared with those in the Sahel affecting the overall higher energy expenditure (Cabello‐Vergel et al., 2021). Finally, we could not record other components of energy balance, such as energy intake, which is likely to differ substantially among individuals overwintering in different areas and with different accessibility to landfill resources.

Notably, our results showed that smaller sized individuals are more likely to migrate than larger sized ones, a pattern that is highly consistent over time (i.e., birds used the same wintering grounds every year). However, given the correlational nature of the analysis, we could not establish a direct causality between size and migratory strategy. Nevertheless, several hypotheses could explain these behavioral differences. Smaller birds may be outcompeted at landfill sites, as in these areas birds gather in large numbers that exacerbates competition and aggression (Gilbert et al., 2016; Soriano‐Redondo et al., 2021). An alternative, nonexclusive explanation is that smaller individuals are more sensitive to harsher wintering conditions in Southwest Europe and migrate to warmer areas in the Sahel.

Our results suggest that differential fitness between migratory and resident birds is likely to exist and might have influenced the recent increase in the ratio of resident to migratory individuals in the population (Catry et al., 2017). As larger birds tend to be residents, occupy the nest earlier and thus are more likely to reproduce, this could be favoring an overall increase in body size in the population, potentially increasing the prevalence of residency. However, the fast‐ongoing population transition toward full residency suggests that other factors may be involved as well. We did not find differences in survival between residents and migrants, but this should be further investigated with larger sample sizes, as the probability of recapture when the GPS signal had been lost was much lower for migrants than residents, and could partially mask the effects on survival.

The availability of landfill waste in Portugal and Spain is expected to decrease substantially in the next few years, as recent EU directives (1999/31/UE and 2018/850/UE) regulating waste disposal have established a reduction of municipal waste landfilled to 10% in the next decade. A dramatic decrease in food availability in the main European wintering areas can have unforeseen consequences for white stork populations. Yet, based on our findings, we predicted an increase in migratory propensity, with only larger individuals being able to remain on the breeding grounds throughout the year. Carry‐over effects may include increased mortality and reduced reproduction success, which could slow down the current increase in population numbers and might even lead to a decrease in population size. Our results highlight the nuances of anthropogenic impacts on species behavior, fitness, and evolutionary dynamics.

## AUTHOR CONTRIBUTIONS

Andrea Soriano‐Redondo, Aldina M. A. Franco and Inês Catry designed the study. Andrea Soriano‐Redondo and Ana Payo‐Payo performed the analyses. Aldina M. A. Franco, Marta Acácio, Bruno Herlander Martins and Inês Catry collected data. Andrea Soriano‐Redondo wrote the first draft of the manuscript, and all authors contributed substantially to revisions.

## CONFLICT OF INTEREST STATEMENT

The authors declare no conflicts of interest.

## Supporting information


Appendix S1.

## Data Availability

Tracking data (Soriano‐Redondo et al., 2023) are available from Movebank at https://doi.org/10.5441/001/1.283.
