# Supplementary material for: Fitness, behavioral, and energetic trade‐offs of different migratory strategies in a partially migratory species
Source: Ecology. 2023 Sep 7;104(10):e4151. doi: 10.1002/ecy.4151 (PMC10909454; doi:10.1002/ecy.4151)
Supplement: Supplementary file 1 — Appendix S1. [file ECY-104-e4151-s001.pdf]

Fitness, behavioral, and energetic trade-offs of different migratory strategies in a partially migratory species

A. Soriano-Redondo; A.M.A. Franco; M. Acácio; A. Payo-Payo; B.H. Martins; F. Moreira; I. Catry

Ecology

Section S1: Annual level

Table S1: Tukey's contrasts multiple comparisons with adjusted p-values for the annual displacement per migratory strategy.

	Estimate	Std. Error	z value	Pr(>|z|)
Regional - Local	219.626	471.848	0.465	1
NW Africa - Local	893.473	934.021	0.957	1
Sub-Sahara - Local	9553.704	644.301	14.828	0
NW Africa - Regional	673.847	855.744	0.787	1
Sub-Sahara - Regional	9334.078	540.438	17.271	0
Sub-Sahara - NW Africa	8660.231	971.981	8.910	0

Table S2: Tukey's contrasts multiple comparisons with adjusted p-values for the annual mean ODBA per migratory strategy.

	Estimate	Std. Error	z value	Pr(>|z|)
Regional - Local	0.004	0.004	1.048	0.835
NW Africa - Local	0.006	0.006	1.084	0.835
Sub-Sahara - Local	0.028	0.005	5.492	0.000
NW Africa - Regional	0.002	0.005	0.473	0.835
Sub-Sahara - Regional	0.024	0.004	5.594	0.000
Sub-Sahara - NW Africa	0.022	0.006	3.480	0.002

Table S3: Tukey's contrasts multiple comparisons with adjusted p-values for the annual mean foraging ODBA per migratory strategy.

	Estimate	Std. Error	z value	Pr(>|z|)
Regional - Local	0.006	0.004	1.467	0.427
NW Africa - Local	0.006	0.006	1.030	0.606
Sub-Sahara - Local	0.024	0.005	4.616	0.000
NW Africa - Regional	0.000	0.005	0.089	0.929
Sub-Sahara - Regional	0.018	0.004	4.199	0.000
Sub-Sahara - NW Africa	0.018	0.006	2.880	0.016

Table S4: Tukey's contrasts multiple comparisons with adjusted p-values for the annual mean soaring ODBA per migratory strategy.

	Estimate	Std. Error	z value	Pr(>|z|)
Regional - Local	-0.009	0.004	-2.011	0.133
NW Africa - Local	-0.012	0.007	-1.690	0.182
Sub-Sahara - Local	-0.049	0.006	-7.969	0.000
NW Africa - Regional	-0.003	0.007	-0.523	0.601
Sub-Sahara - Regional	-0.040	0.005	-7.563	0.000
Sub-Sahara - NW Africa	-0.036	0.008	-4.615	0.000

Table S5: Tukey's contrasts multiple comparisons with adjusted p-values for the proportion of resting time per migratory strategy.

	Estimate	Std. Error	z value	Pr(>|z|)
Regional - Local	-0.094	0.083	-1.125	0.642
NW Africa - Local	-0.162	0.131	-1.243	0.642
Sub-Sahara - Local	-0.661	0.115	-5.750	0.000
NW Africa - Regional	-0.069	0.112	-0.613	0.642
Sub-Sahara - Regional	-0.568	0.096	-5.938	0.000
Sub-Sahara - NW Africa	-0.499	0.139	-3.601	0.001

Table S6: Tukey's contrasts multiple comparisons with adjusted p-values for the proportion of soaring time per migratory strategy.

	Estimate	Std. Error	z value	Pr(>|z|)
Regional - Local	0.184	0.074	2.493	0.013
NW Africa - Local	0.466	0.112	4.166	0.000
Sub-Sahara - Local	1.188	0.092	12.962	0.000
NW Africa - Regional	0.283	0.095	2.967	0.006
Sub-Sahara - Regional	1.005	0.072	13.976	0.000
Sub-Sahara - NW Africa	0.722	0.110	6.561	0.000

Section S2: Seasonal level

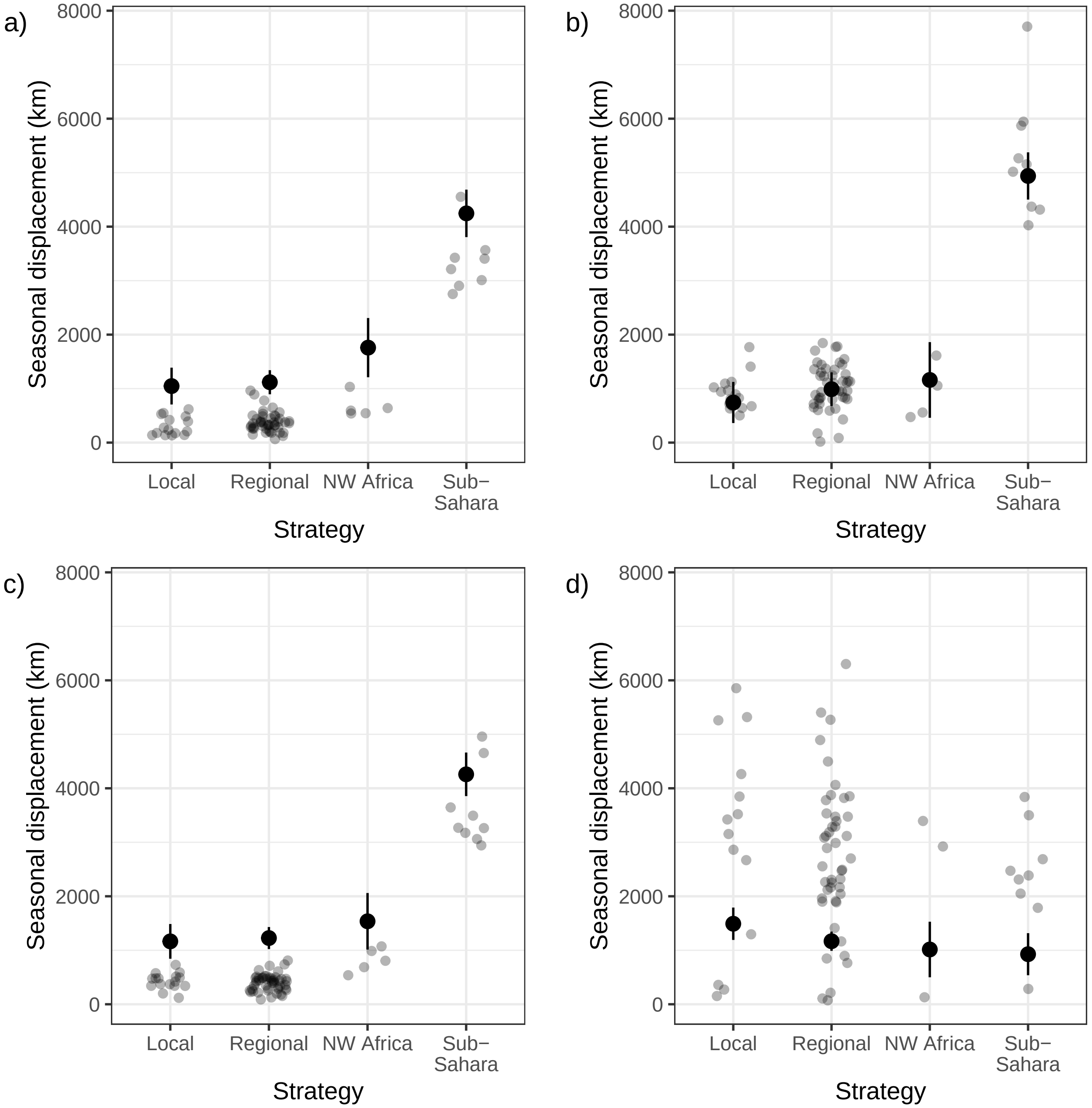

Fig S1: Relationship between the migratory strategy and the seasonal displacement, during the (a) autumn transition, (b) the wintering period, (c) the spring transition, and (d) the breeding period. Black dots are predicted estimates from the LMM, vertical lines are the confidence intervals based on fixed-effect uncertainty, and grey dots are raw data.

Table S7: Tukey's contrasts multiple comparisons with adjusted p-values for the seasonal displacement per migratory strategy and season.

contrast	estimate	SE	df	t.ratio	p.value
Local Autumn - Regional Autumn	-71.326	179.605	232.244	-0.397	1.000
Local Autumn - NW Africa Autumn	-712.123	306.928	270.033	-2.320	0.609
Local Autumn - (Sub-Sahara Autumn)	-3198.770	260.759	257.930	-12.267	0.000

Table S7 *(continued)*

contrast	estimate	SE	df	t.ratio	p.value
Local Autumn - Local Breeding	-444.107	275.995	266.860	-1.609	0.966
Local Autumn - Regional Breeding	-120.529	275.938	268.344	-0.437	1.000
Local Autumn - NW Africa Breeding	32.713	421.540	280.524	0.078	1.000
Local Autumn - (Sub-Sahara Breeding)	119.816	308.636	263.031	0.388	1.000
Local Autumn - Local Spring	-117.685	204.053	236.351	-0.577	1.000
Local Autumn - Regional Spring	-179.312	182.854	234.880	-0.981	1.000
Local Autumn - NW Africa Spring	-489.661	307.816	270.263	-1.591	0.969
Local Autumn - (Sub-Sahara Spring)	-3211.842	253.163	246.962	-12.687	0.000
Local Autumn - Local Wintering	303.782	220.905	247.136	1.375	0.992
Local Autumn - Regional Wintering	57.322	205.866	244.653	0.278	1.000
Local Autumn - NW Africa Wintering	-113.548	312.529	269.641	-0.363	1.000
Local Autumn - (Sub-Sahara Wintering)	-3891.896	269.943	252.160	-14.417	0.000
Regional Autumn - NW Africa Autumn	-640.797	277.212	280.948	-2.312	0.615
Regional Autumn - (Sub-Sahara Autumn)	-3127.445	225.415	272.114	-13.874	0.000
Regional Autumn - Local Breeding	-372.781	255.363	276.779	-1.460	0.986
Regional Autumn - Regional Breeding	-49.203	237.495	281.074	-0.207	1.000
Regional Autumn - NW Africa Breeding	104.038	398.895	284.706	0.261	1.000
Regional Autumn - (Sub-Sahara Breeding)	191.141	277.901	277.848	0.688	1.000
Regional Autumn - Local Spring	-46.359	177.100	222.482	-0.262	1.000
Regional Autumn - Regional Spring	-107.986	120.585	245.379	-0.896	1.000
Regional Autumn - NW Africa Spring	-418.335	277.735	281.306	-1.506	0.981
Regional Autumn - (Sub-Sahara Spring)	-3140.516	216.268	261.861	-14.521	0.000
Regional Autumn - Local Wintering	375.108	195.443	247.719	1.919	0.865
Regional Autumn - Regional Wintering	128.648	152.067	269.401	0.846	1.000
Regional Autumn - NW Africa Wintering	-42.223	282.668	281.439	-0.149	1.000
Regional Autumn - (Sub-Sahara Wintering)	-3820.570	234.939	269.570	-16.262	0.000
NW Africa Autumn - (Sub-Sahara Autumn)	-2486.647	334.998	278.225	-7.423	0.000
NW Africa Autumn - Local Breeding	268.017	368.974	280.563	0.726	1.000
NW Africa Autumn - Regional Breeding	591.594	360.651	283.614	1.640	0.959
NW Africa Autumn - NW Africa Breeding	744.836	469.267	266.246	1.587	0.969
NW Africa Autumn - (Sub-Sahara Breeding)	831.939	384.718	280.957	2.162	0.722
NW Africa Autumn - Local Spring	594.438	306.562	267.377	1.939	0.855
NW Africa Autumn - Regional Spring	532.811	280.489	279.546	1.900	0.874
NW Africa Autumn - NW Africa Spring	222.462	359.735	234.271	0.618	1.000
NW Africa Autumn - (Sub-Sahara Spring)	-2499.719	330.819	275.389	-7.556	0.000
NW Africa Autumn - Local Wintering	1015.906	323.227	272.944	3.143	0.123
NW Africa Autumn - Regional Wintering	769.445	301.786	280.707	2.550	0.439
NW Africa Autumn - NW Africa Wintering	598.575	366.193	236.766	1.635	0.960
NW Africa Autumn - (Sub-Sahara Wintering)	-3179.773	349.081	278.055	-9.109	0.000
(Sub-Sahara Autumn) - Local Breeding	2754.664	324.078	276.965	8.500	0.000
(Sub-Sahara Autumn) - Regional Breeding	3078.241	313.780	281.194	9.810	0.000
(Sub-Sahara Autumn) - NW Africa Breeding	3231.483	446.797	283.253	7.233	0.000
(Sub-Sahara Autumn) - (Sub-Sahara Breeding)	3318.586	331.568	256.325	10.009	0.000
(Sub-Sahara Autumn) - Local Spring	3081.085	259.692	253.740	11.864	0.000
(Sub-Sahara Autumn) - Regional Spring	3019.458	228.616	270.048	13.208	0.000
(Sub-Sahara Autumn) - NW Africa Spring	2709.109	337.048	278.335	8.038	0.000
(Sub-Sahara Autumn) - (Sub-Sahara Spring)	-13.071	275.340	235.294	-0.047	1.000
(Sub-Sahara Autumn) - Local Wintering	3502.553	275.567	263.191	12.710	0.000

Table S7 *(continued)*

contrast	estimate	SE	df	t.ratio	p.value
(Sub-Sahara Autumn) - Regional Wintering	3256.092	250.200	273.578	13.014	0.000
(Sub-Sahara Autumn) - NW Africa Wintering	3085.222	342.387	278.722	9.011	0.000
(Sub-Sahara Autumn) - (Sub-Sahara Wintering)	-693.125	293.337	243.200	-2.363	0.577
Local Breeding - Regional Breeding	323.577	189.785	240.171	1.705	0.944
Local Breeding - NW Africa Breeding	476.819	379.330	282.008	1.257	0.997
Local Breeding - (Sub-Sahara Breeding)	563.922	255.812	248.123	2.204	0.693
Local Breeding - Local Spring	326.422	266.374	265.623	1.225	0.998
Local Breeding - Regional Spring	264.794	247.965	275.896	1.068	1.000
Local Breeding - NW Africa Spring	-45.554	347.538	279.057	-0.131	1.000
Local Breeding - (Sub-Sahara Spring)	-2767.735	303.898	269.081	-9.107	0.000
Local Breeding - Local Wintering	747.889	227.976	251.460	3.281	0.085
Local Breeding - Regional Wintering	501.428	208.304	260.295	2.407	0.544
Local Breeding - NW Africa Wintering	330.558	331.421	276.842	0.997	1.000
Local Breeding - (Sub-Sahara Wintering)	-3447.789	269.431	257.265	-12.797	0.000
Regional Breeding - NW Africa Breeding	153.242	349.801	284.997	0.438	1.000
Regional Breeding - (Sub-Sahara Breeding)	240.345	220.056	259.591	1.092	0.999
Regional Breeding - Local Spring	2.844	262.378	263.513	0.011	1.000
Regional Breeding - Regional Spring	-58.783	223.016	279.103	-0.264	1.000
Regional Breeding - NW Africa Spring	-369.132	332.533	283.291	-1.110	0.999
Regional Breeding - (Sub-Sahara Spring)	-3091.312	289.316	275.524	-10.685	0.000
Regional Breeding - Local Wintering	424.312	214.052	245.690	1.982	0.833
Regional Breeding - Regional Wintering	177.851	163.654	268.919	1.087	0.999
Regional Breeding - NW Africa Wintering	6.981	312.932	282.558	0.022	1.000
Regional Breeding - (Sub-Sahara Wintering)	-3771.366	244.265	267.317	-15.440	0.000
NW Africa Breeding - (Sub-Sahara Breeding)	87.103	394.933	282.491	0.221	1.000
NW Africa Breeding - Local Spring	-150.397	412.250	280.024	-0.365	1.000
NW Africa Breeding - Regional Spring	-212.025	389.442	284.770	-0.544	1.000
NW Africa Breeding - NW Africa Spring	-522.373	448.629	261.686	-1.164	0.999
NW Africa Breeding - (Sub-Sahara Spring)	-3244.554	430.455	282.066	-7.538	0.000
NW Africa Breeding - Local Wintering	271.070	386.581	280.195	0.701	1.000
NW Africa Breeding - Regional Wintering	24.609	361.828	284.841	0.068	1.000
NW Africa Breeding - NW Africa Wintering	-146.261	436.129	258.875	-0.335	1.000
NW Africa Breeding - (Sub-Sahara Wintering)	-3924.608	405.072	282.033	-9.689	0.000
(Sub-Sahara Breeding) - Local Spring	-237.501	299.908	257.619	-0.792	1.000
(Sub-Sahara Breeding) - Regional Spring	-299.128	270.686	275.845	-1.105	0.999
(Sub-Sahara Breeding) - NW Africa Spring	-609.477	364.110	279.505	-1.674	0.952
(Sub-Sahara Breeding) - (Sub-Sahara Spring)	-3331.657	311.086	253.611	-10.710	0.000
(Sub-Sahara Breeding) - Local Wintering	183.967	266.824	245.793	0.689	1.000
(Sub-Sahara Breeding) - Regional Wintering	-62.494	235.221	265.749	-0.266	1.000
(Sub-Sahara Breeding) - NW Africa Wintering	-233.364	349.016	278.186	-0.669	1.000
(Sub-Sahara Breeding) - (Sub-Sahara Wintering)	-4011.711	278.001	239.517	-14.431	0.000
Local Spring - Regional Spring	-61.627	175.680	225.605	-0.351	1.000
Local Spring - NW Africa Spring	-371.976	303.945	267.783	-1.224	0.998
Local Spring - (Sub-Sahara Spring)	-3094.157	249.954	241.495	-12.379	0.000
Local Spring - Local Wintering	421.467	212.034	241.744	1.988	0.830
Local Spring - Regional Wintering	175.007	193.639	234.489	0.904	1.000
Local Spring - NW Africa Wintering	4.137	307.572	266.630	0.013	1.000
Local Spring - (Sub-Sahara Wintering)	-3774.211	263.316	245.529	-14.333	0.000

Table S7 *(continued)*

contrast	estimate	SE	df	t.ratio	p.value
Regional Spring - NW Africa Spring	-310.349	276.010	280.419	-1.124	0.999
Regional Spring - (Sub-Sahara Spring)	-3032.529	216.498	259.374	-14.007	0.000
Regional Spring - Local Wintering	483.095	188.731	247.110	2.560	0.432
Regional Spring - Regional Wintering	236.634	137.951	255.867	1.715	0.941
Regional Spring - NW Africa Wintering	65.764	279.713	280.368	0.235	1.000
Regional Spring - (Sub-Sahara Wintering)	-3712.583	230.543	266.476	-16.104	0.000
NW Africa Spring - (Sub-Sahara Spring)	-2722.181	328.795	275.099	-8.279	0.000
NW Africa Spring - Local Wintering	793.443	310.378	271.490	2.556	0.434
NW Africa Spring - Regional Wintering	546.983	285.182	280.756	1.918	0.866
NW Africa Spring - NW Africa Wintering	376.113	358.258	233.271	1.050	1.000
NW Africa Spring - (Sub-Sahara Wintering)	-3402.235	336.862	276.718	-10.100	0.000
(Sub-Sahara Spring) - Local Wintering	3515.624	259.565	249.786	13.544	0.000
(Sub-Sahara Spring) - Regional Wintering	3269.164	230.805	262.223	14.164	0.000
(Sub-Sahara Spring) - NW Africa Wintering	3098.293	331.602	275.033	9.343	0.000
(Sub-Sahara Spring) - (Sub-Sahara Wintering)	-680.054	277.131	238.935	-2.454	0.509
Local Wintering - Regional Wintering	-246.461	176.654	227.177	-1.395	0.991
Local Wintering - NW Africa Wintering	-417.331	304.824	268.603	-1.369	0.993
Local Wintering - (Sub-Sahara Wintering)	-4195.678	250.213	241.023	-16.768	0.000
Regional Wintering - NW Africa Wintering	-170.870	278.688	279.893	-0.613	1.000
Regional Wintering - (Sub-Sahara Wintering)	-3949.218	218.027	258.449	-18.113	0.000
NW Africa Wintering - (Sub-Sahara Wintering)	-3778.347	330.388	275.651	-11.436	0.000

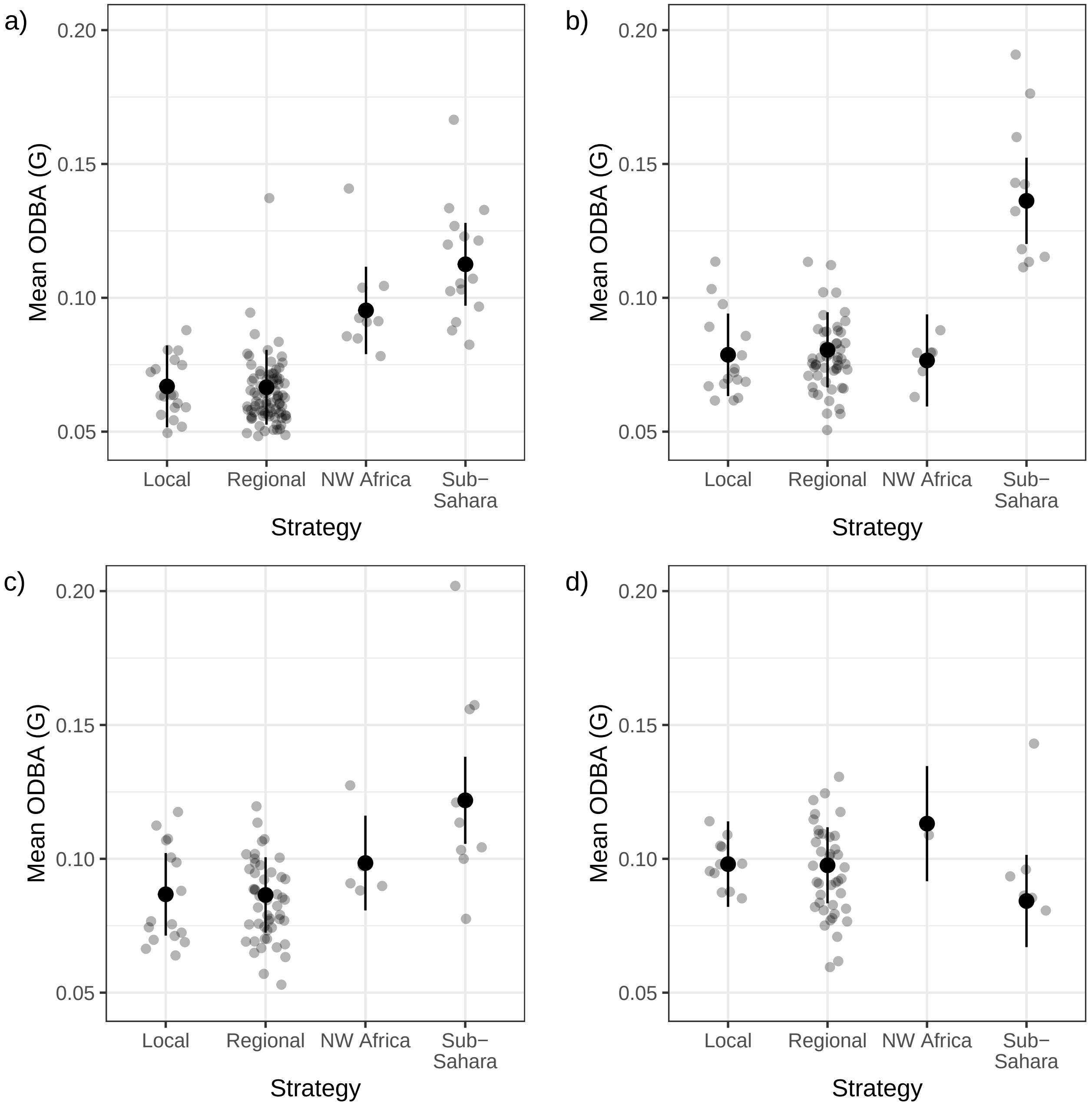

Fig S2: Relationship between the migratory strategy and the seasonal mean ODBA, during the (a) autumn transition, (b) the wintering period, (c) the spring transition, and (d) the breeding period. Black dots are predicted estimates from the LMM, vertical lines are the confidence intervals based on fixed-effect uncertainty, and grey dots are raw data.

Table S8: Tukey's contrasts multiple comparisons with adjusted p-values for the seasonal mean ODBA per migratory strategy and season.

contrast	estimate	SE	df	t.ratio	p.value
Local Autumn - Regional Autumn	0.000	0.004	235.183	0.081	1.000
Local Autumn - NW Africa Autumn	-0.028	0.006	226.409	-5.021	0.000
Local Autumn - (Sub-Sahara Autumn)	-0.046	0.005	171.283	-8.926	0.000
Local Autumn - Local Wintering	-0.012	0.004	266.898	-3.133	0.126
Local Autumn - Regional Wintering	-0.014	0.004	244.203	-3.485	0.047

Table S8 *(continued)*

contrast	estimate	SE	df	t.ratio	p.value
Local Autumn - NW Africa Wintering	-0.010	0.006	266.038	-1.544	0.976
Local Autumn - (Sub-Sahara Wintering)	-0.069	0.006	198.589	-12.393	0.000
Local Autumn - Local Spring	-0.020	0.004	266.898	-5.260	0.000
Local Autumn - Regional Spring	-0.020	0.004	245.201	-4.986	0.000
Local Autumn - NW Africa Spring	-0.032	0.007	284.689	-4.740	0.000
Local Autumn - (Sub-Sahara Spring)	-0.055	0.006	208.947	-9.630	0.000
Local Autumn - Local Breeding	-0.031	0.004	271.150	-7.308	0.000
Local Autumn - Regional Breeding	-0.031	0.004	252.419	-7.682	0.000
Local Autumn - NW Africa Breeding	-0.046	0.009	323.592	-5.066	0.000
Local Autumn - (Sub-Sahara Breeding)	-0.017	0.006	251.743	-2.717	0.326
Regional Autumn - NW Africa Autumn	-0.029	0.005	238.093	-6.009	0.000
Regional Autumn - (Sub-Sahara Autumn)	-0.046	0.004	146.767	-11.108	0.000
Regional Autumn - Local Wintering	-0.012	0.004	252.179	-3.136	0.126
Regional Autumn - Regional Wintering	-0.014	0.002	268.313	-7.073	0.000
Regional Autumn - NW Africa Wintering	-0.010	0.006	286.656	-1.821	0.907
Regional Autumn - (Sub-Sahara Wintering)	-0.070	0.005	188.242	-14.744	0.000
Regional Autumn - Local Spring	-0.020	0.004	252.179	-5.213	0.000
Regional Autumn - Regional Spring	-0.020	0.002	269.094	-9.947	0.000
Regional Autumn - NW Africa Spring	-0.032	0.006	305.133	-5.391	0.000
Regional Autumn - (Sub-Sahara Spring)	-0.055	0.005	202.854	-11.380	0.000
Regional Autumn - Local Breeding	-0.031	0.004	289.701	-7.117	0.000
Regional Autumn - Regional Breeding	-0.031	0.002	268.789	-14.591	0.000
Regional Autumn - NW Africa Breeding	-0.047	0.009	313.860	-5.433	0.000
Regional Autumn - (Sub-Sahara Breeding)	-0.018	0.006	261.413	-3.122	0.130
NW Africa Autumn - (Sub-Sahara Autumn)	-0.017	0.006	195.035	-2.926	0.212
NW Africa Autumn - Local Wintering	0.017	0.006	233.605	2.882	0.232
NW Africa Autumn - Regional Wintering	0.015	0.005	249.008	3.013	0.171
NW Africa Autumn - NW Africa Wintering	0.019	0.006	281.471	3.174	0.113
NW Africa Autumn - (Sub-Sahara Wintering)	-0.041	0.006	214.899	-6.486	0.000
NW Africa Autumn - Local Spring	0.009	0.006	233.605	1.486	0.983
NW Africa Autumn - Regional Spring	0.009	0.005	249.681	1.795	0.916
NW Africa Autumn - NW Africa Spring	-0.003	0.006	285.031	-0.500	1.000
NW Africa Autumn - (Sub-Sahara Spring)	-0.027	0.006	222.960	-4.142	0.005
NW Africa Autumn - Local Breeding	-0.003	0.006	255.969	-0.446	1.000
NW Africa Autumn - Regional Breeding	-0.002	0.005	254.348	-0.463	1.000
NW Africa Autumn - NW Africa Breeding	-0.018	0.009	282.720	-2.009	0.819
NW Africa Autumn - (Sub-Sahara Breeding)	0.011	0.007	256.539	1.574	0.972
(Sub-Sahara Autumn) - Local Wintering	0.034	0.005	180.446	6.481	0.000
(Sub-Sahara Autumn) - Regional Wintering	0.032	0.004	159.616	7.483	0.000
(Sub-Sahara Autumn) - NW Africa Wintering	0.036	0.007	235.798	5.517	0.000
(Sub-Sahara Autumn) - (Sub-Sahara Wintering)	-0.024	0.005	280.220	-5.200	0.000
(Sub-Sahara Autumn) - Local Spring	0.026	0.005	180.446	4.942	0.000
(Sub-Sahara Autumn) - Regional Spring	0.026	0.004	160.452	6.077	0.000
(Sub-Sahara Autumn) - NW Africa Spring	0.014	0.007	257.367	2.056	0.791
(Sub-Sahara Autumn) - (Sub-Sahara Spring)	-0.009	0.005	279.692	-1.982	0.833
(Sub-Sahara Autumn) - Local Breeding	0.014	0.006	211.951	2.570	0.425
(Sub-Sahara Autumn) - Regional Breeding	0.015	0.004	167.018	3.435	0.057
(Sub-Sahara Autumn) - NW Africa Breeding	-0.001	0.009	325.092	-0.067	1.000

Table S8 *(continued)*

contrast	estimate	SE	df	t.ratio	p.value
(Sub-Sahara Autumn) - (Sub-Sahara Breeding)	0.028	0.005	280.206	5.184	0.000
Local Wintering - Regional Wintering	-0.002	0.004	259.129	-0.452	1.000
Local Wintering - NW Africa Wintering	0.002	0.006	270.809	0.330	1.000
Local Wintering - (Sub-Sahara Wintering)	-0.058	0.006	206.218	-10.107	0.000
Local Wintering - Local Spring	-0.008	0.004	261.493	-2.081	0.775
Local Wintering - Regional Spring	-0.008	0.004	259.945	-1.912	0.868
Local Wintering - NW Africa Spring	-0.020	0.007	288.133	-2.927	0.209
Local Wintering - (Sub-Sahara Spring)	-0.043	0.006	216.131	-7.436	0.000
Local Wintering - Local Breeding	-0.019	0.004	267.995	-4.453	0.001
Local Wintering - Regional Breeding	-0.019	0.004	266.144	-4.570	0.001
Local Wintering - NW Africa Breeding	-0.034	0.009	323.376	-3.746	0.019
Local Wintering - (Sub-Sahara Breeding)	-0.006	0.006	256.436	-0.854	1.000
Regional Wintering - NW Africa Wintering	0.004	0.006	292.404	0.704	1.000
Regional Wintering - (Sub-Sahara Wintering)	-0.056	0.005	198.603	-11.492	0.000
Regional Wintering - Local Spring	-0.006	0.004	259.129	-1.527	0.978
Regional Wintering - Regional Spring	-0.006	0.002	263.144	-2.687	0.344
Regional Wintering - NW Africa Spring	-0.018	0.006	308.675	-2.981	0.183
Regional Wintering - (Sub-Sahara Spring)	-0.041	0.005	212.459	-8.302	0.000
Regional Wintering - Local Breeding	-0.017	0.005	291.927	-3.806	0.016
Regional Wintering - Regional Breeding	-0.017	0.002	266.161	-7.271	0.000
Regional Wintering - NW Africa Breeding	-0.033	0.009	313.264	-3.779	0.017
Regional Wintering - (Sub-Sahara Breeding)	-0.004	0.006	267.325	-0.641	1.000
NW Africa Wintering - (Sub-Sahara Wintering)	-0.060	0.007	249.013	-8.647	0.000
NW Africa Wintering - Local Spring	-0.010	0.006	270.809	-1.588	0.969
NW Africa Wintering - Regional Spring	-0.010	0.006	292.713	-1.762	0.927
NW Africa Wintering - NW Africa Spring	-0.022	0.007	269.653	-3.265	0.088
NW Africa Wintering - (Sub-Sahara Spring)	-0.045	0.007	255.142	-6.476	0.000
NW Africa Wintering - Local Breeding	-0.021	0.007	284.595	-3.181	0.111
NW Africa Wintering - Regional Breeding	-0.021	0.006	295.047	-3.710	0.022
NW Africa Wintering - NW Africa Breeding	-0.037	0.009	282.291	-3.933	0.010
NW Africa Wintering - (Sub-Sahara Breeding)	-0.008	0.008	279.083	-1.010	1.000
(Sub-Sahara Wintering) - Local Spring	0.049	0.006	206.218	8.696	0.000
(Sub-Sahara Wintering) - Regional Spring	0.050	0.005	199.215	10.246	0.000
(Sub-Sahara Wintering) - NW Africa Spring	0.038	0.007	267.062	5.235	0.000
(Sub-Sahara Wintering) - (Sub-Sahara Spring)	0.014	0.005	262.567	2.860	0.243
(Sub-Sahara Wintering) - Local Breeding	0.038	0.006	231.775	6.283	0.000
(Sub-Sahara Wintering) - Regional Breeding	0.039	0.005	204.254	7.868	0.000
(Sub-Sahara Wintering) - NW Africa Breeding	0.023	0.010	325.007	2.416	0.537
(Sub-Sahara Wintering) - (Sub-Sahara Breeding)	0.052	0.006	270.782	9.035	0.000
Local Spring - Regional Spring	0.000	0.004	259.945	0.062	1.000
Local Spring - NW Africa Spring	-0.012	0.007	288.133	-1.735	0.936
Local Spring - (Sub-Sahara Spring)	-0.035	0.006	216.131	-6.052	0.000
Local Spring - Local Breeding	-0.011	0.004	267.995	-2.602	0.402
Local Spring - Regional Breeding	-0.011	0.004	266.144	-2.625	0.386
Local Spring - NW Africa Breeding	-0.026	0.009	323.376	-2.872	0.235
Local Spring - (Sub-Sahara Breeding)	0.003	0.006	256.436	0.387	1.000
Regional Spring - NW Africa Spring	-0.012	0.006	308.827	-1.988	0.830
Regional Spring - (Sub-Sahara Spring)	-0.035	0.005	212.995	-7.094	0.000

Table S8 *(continued)*

contrast	estimate	SE	df	t.ratio	p.value
Regional Spring - Local Breeding	-0.012	0.005	292.292	-2.507	0.470
Regional Spring - Regional Breeding	-0.011	0.002	265.283	-4.708	0.000
Regional Spring - NW Africa Breeding	-0.027	0.009	313.196	-3.088	0.141
Regional Spring - (Sub-Sahara Breeding)	0.002	0.006	267.550	0.390	1.000
NW Africa Spring - (Sub-Sahara Spring)	-0.023	0.007	272.013	-3.204	0.104
NW Africa Spring - Local Breeding	0.000	0.007	297.822	0.056	1.000
NW Africa Spring - Regional Breeding	0.001	0.006	310.188	0.140	1.000
NW Africa Spring - NW Africa Breeding	-0.015	0.009	279.425	-1.554	0.975
NW Africa Spring - (Sub-Sahara Breeding)	0.014	0.008	290.572	1.807	0.912
(Sub-Sahara Spring) - Local Breeding	0.024	0.006	239.996	3.852	0.014
(Sub-Sahara Spring) - Regional Breeding	0.024	0.005	217.676	4.815	0.000
(Sub-Sahara Spring) - NW Africa Breeding	0.009	0.010	325.144	0.905	1.000
(Sub-Sahara Spring) - (Sub-Sahara Breeding)	0.038	0.006	271.368	6.403	0.000
Local Breeding - Regional Breeding	0.000	0.005	295.660	0.096	1.000
Local Breeding - NW Africa Breeding	-0.015	0.009	322.919	-1.601	0.967
Local Breeding - (Sub-Sahara Breeding)	0.014	0.007	271.808	2.024	0.810
Regional Breeding - NW Africa Breeding	-0.016	0.009	312.857	-1.797	0.916
Regional Breeding - (Sub-Sahara Breeding)	0.013	0.006	270.000	2.294	0.628
NW Africa Breeding - (Sub-Sahara Breeding)	0.029	0.010	325.188	2.877	0.233

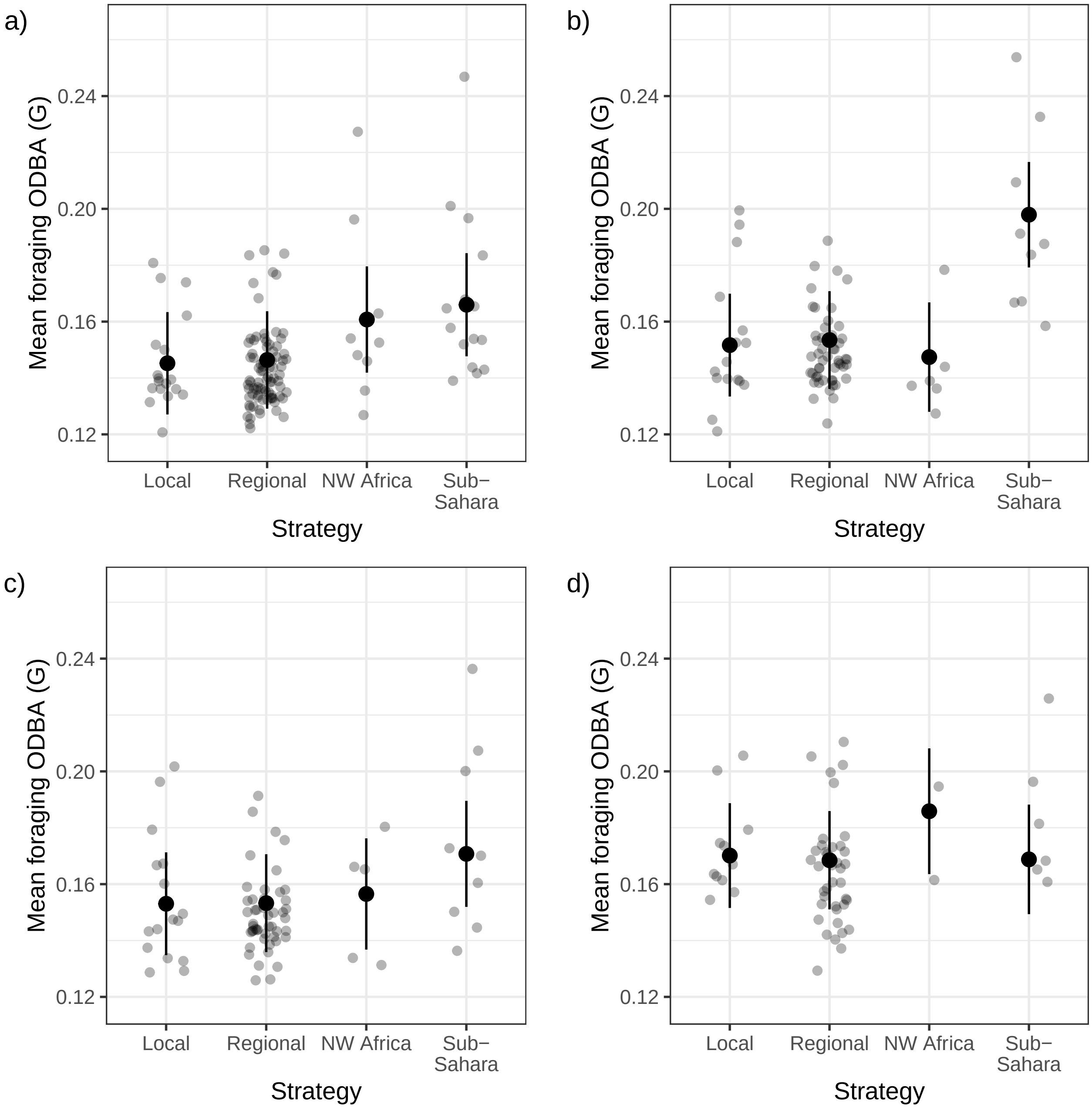

Fig S3: Relationship between the migratory strategy and the seasonal mean foraging ODBA, during the (a) autumn transition, (b) the wintering period, (c) the spring transition, and (d) the breeding period. Black dots are predicted estimates from the LMM, vertical lines are the confidence intervals based on fixed-effect uncertainty, and grey dots are raw data.

Table S9: Tukey's contrasts multiple comparisons with adjusted p-values for the seasonal mean foraging ODBA per migratory strategy and season.

contrast	estimate	SE	df	t.ratio	p.value
Local Autumn - Regional Autumn	-0.001	0.003	239.486	-0.356	1.000
Local Autumn - NW Africa Autumn	-0.016	0.005	222.079	-3.097	0.140
Local Autumn - (Sub-Sahara Autumn)	-0.021	0.005	161.681	-4.542	0.001
Local Autumn - Local Wintering	-0.006	0.003	266.014	-1.998	0.824
Local Autumn - Regional Wintering	-0.008	0.003	248.102	-2.381	0.564

Table S9 *(continued)*

contrast	estimate	SE	df	t.ratio	p.value
Local Autumn - NW Africa Wintering	-0.002	0.006	263.096	-0.395	1.000
Local Autumn - (Sub-Sahara Wintering)	-0.053	0.005	188.792	-10.586	0.000
Local Autumn - Local Spring	-0.008	0.003	266.014	-2.423	0.532
Local Autumn - Regional Spring	-0.008	0.003	249.142	-2.312	0.615
Local Autumn - NW Africa Spring	-0.011	0.006	282.753	-1.933	0.858
Local Autumn - (Sub-Sahara Spring)	-0.026	0.005	198.244	-5.032	0.000
Local Autumn - Local Breeding	-0.025	0.004	269.403	-6.842	0.000
Local Autumn - Regional Breeding	-0.023	0.004	256.081	-6.602	0.000
Local Autumn - NW Africa Breeding	-0.041	0.008	323.411	-5.147	0.000
Local Autumn - (Sub-Sahara Breeding)	-0.024	0.006	241.713	-4.182	0.004
Regional Autumn - NW Africa Autumn	-0.014	0.004	231.996	-3.396	0.061
Regional Autumn - (Sub-Sahara Autumn)	-0.020	0.004	134.022	-5.269	0.000
Regional Autumn - Local Wintering	-0.005	0.003	256.746	-1.545	0.976
Regional Autumn - Regional Wintering	-0.007	0.002	267.220	-4.190	0.004
Regional Autumn - NW Africa Wintering	-0.001	0.005	283.353	-0.211	1.000
Regional Autumn - (Sub-Sahara Wintering)	-0.052	0.004	174.024	-12.234	0.000
Regional Autumn - Local Spring	-0.007	0.003	256.746	-1.947	0.851
Regional Autumn - Regional Spring	-0.007	0.002	267.916	-4.007	0.008
Regional Autumn - NW Africa Spring	-0.010	0.005	303.499	-1.959	0.845
Regional Autumn - (Sub-Sahara Spring)	-0.024	0.004	187.165	-5.634	0.000
Regional Autumn - Local Breeding	-0.024	0.004	293.187	-6.132	0.000
Regional Autumn - Regional Breeding	-0.022	0.002	267.503	-12.170	0.000
Regional Autumn - NW Africa Breeding	-0.039	0.007	312.745	-5.347	0.000
Regional Autumn - (Sub-Sahara Breeding)	-0.022	0.005	247.333	-4.493	0.001
NW Africa Autumn - (Sub-Sahara Autumn)	-0.005	0.005	182.170	-1.000	1.000
NW Africa Autumn - Local Wintering	0.009	0.005	229.613	1.784	0.920
NW Africa Autumn - Regional Wintering	0.007	0.004	242.783	1.685	0.949
NW Africa Autumn - NW Africa Wintering	0.013	0.005	280.402	2.645	0.372
NW Africa Autumn - (Sub-Sahara Wintering)	-0.037	0.006	202.582	-6.636	0.000
NW Africa Autumn - Local Spring	0.008	0.005	229.613	1.514	0.980
NW Africa Autumn - Regional Spring	0.007	0.004	243.536	1.732	0.936
NW Africa Autumn - NW Africa Spring	0.004	0.005	283.490	0.787	1.000
NW Africa Autumn - (Sub-Sahara Spring)	-0.010	0.006	210.064	-1.758	0.928
NW Africa Autumn - Local Breeding	-0.009	0.005	252.494	-1.736	0.935
NW Africa Autumn - Regional Breeding	-0.008	0.004	248.279	-1.771	0.924
NW Africa Autumn - NW Africa Breeding	-0.025	0.008	280.537	-3.302	0.079
NW Africa Autumn - (Sub-Sahara Breeding)	-0.008	0.006	244.863	-1.299	0.996
(Sub-Sahara Autumn) - Local Wintering	0.014	0.005	170.618	3.076	0.150
(Sub-Sahara Autumn) - Regional Wintering	0.013	0.004	145.512	3.269	0.093
(Sub-Sahara Autumn) - NW Africa Wintering	0.019	0.006	223.405	3.224	0.100
(Sub-Sahara Autumn) - (Sub-Sahara Wintering)	-0.032	0.004	278.989	-8.184	0.000
(Sub-Sahara Autumn) - Local Spring	0.013	0.005	170.618	2.781	0.290
(Sub-Sahara Autumn) - Regional Spring	0.013	0.004	146.342	3.317	0.081
(Sub-Sahara Autumn) - NW Africa Spring	0.009	0.006	246.086	1.567	0.973
(Sub-Sahara Autumn) - (Sub-Sahara Spring)	-0.005	0.004	278.486	-1.180	0.998
(Sub-Sahara Autumn) - Local Breeding	-0.004	0.005	201.463	-0.829	1.000
(Sub-Sahara Autumn) - Regional Breeding	-0.002	0.004	152.370	-0.641	1.000
(Sub-Sahara Autumn) - NW Africa Breeding	-0.020	0.008	324.195	-2.467	0.499

Table S9 *(continued)*

contrast	estimate	SE	df	t.ratio	p.value
(Sub-Sahara Autumn) - (Sub-Sahara Breeding)	-0.003	0.005	278.081	-0.600	1.000
Local Wintering - Regional Wintering	-0.002	0.004	263.353	-0.503	1.000
Local Wintering - NW Africa Wintering	0.004	0.006	268.308	0.757	1.000
Local Wintering - (Sub-Sahara Wintering)	-0.046	0.005	196.521	-9.148	0.000
Local Wintering - Local Spring	-0.001	0.003	260.755	-0.416	1.000
Local Wintering - Regional Spring	-0.002	0.004	264.214	-0.442	1.000
Local Wintering - NW Africa Spring	-0.005	0.006	286.602	-0.821	1.000
Local Wintering - (Sub-Sahara Spring)	-0.019	0.005	205.641	-3.706	0.024
Local Wintering - Local Breeding	-0.018	0.004	266.342	-4.986	0.000
Local Wintering - Regional Breeding	-0.017	0.004	270.145	-4.629	0.001
Local Wintering - NW Africa Breeding	-0.034	0.008	323.119	-4.303	0.002
Local Wintering - (Sub-Sahara Breeding)	-0.017	0.006	246.953	-3.002	0.176
Regional Wintering - NW Africa Wintering	0.006	0.005	289.207	1.232	0.998
Regional Wintering - (Sub-Sahara Wintering)	-0.044	0.004	183.767	-10.308	0.000
Regional Wintering - Local Spring	0.000	0.004	263.353	0.119	1.000
Regional Wintering - Regional Spring	0.000	0.002	262.239	0.114	1.000
Regional Wintering - NW Africa Spring	-0.003	0.005	307.107	-0.582	1.000
Regional Wintering - (Sub-Sahara Spring)	-0.017	0.004	196.387	-3.909	0.012
Regional Wintering - Local Breeding	-0.017	0.004	295.318	-4.144	0.004
Regional Wintering - Regional Breeding	-0.015	0.002	264.789	-7.509	0.000
Regional Wintering - NW Africa Breeding	-0.032	0.007	312.101	-4.361	0.002
Regional Wintering - (Sub-Sahara Breeding)	-0.015	0.005	253.524	-3.022	0.168
NW Africa Wintering - (Sub-Sahara Wintering)	-0.051	0.006	237.980	-8.297	0.000
NW Africa Wintering - Local Spring	-0.006	0.006	268.308	-1.001	1.000
NW Africa Wintering - Regional Spring	-0.006	0.005	289.594	-1.187	0.998
NW Africa Wintering - NW Africa Spring	-0.009	0.006	268.294	-1.594	0.968
NW Africa Wintering - (Sub-Sahara Spring)	-0.023	0.006	243.913	-3.785	0.018
NW Africa Wintering - Local Breeding	-0.023	0.006	282.819	-3.845	0.014
NW Africa Wintering - Regional Breeding	-0.021	0.005	292.093	-4.254	0.003
NW Africa Wintering - NW Africa Breeding	-0.038	0.008	279.575	-4.836	0.000
NW Africa Wintering - (Sub-Sahara Breeding)	-0.021	0.007	269.949	-3.220	0.100
(Sub-Sahara Wintering) - Local Spring	0.045	0.005	196.521	8.877	0.000
(Sub-Sahara Wintering) - Regional Spring	0.045	0.004	184.441	10.339	0.000
(Sub-Sahara Wintering) - NW Africa Spring	0.041	0.006	257.326	6.513	0.000
(Sub-Sahara Wintering) - (Sub-Sahara Spring)	0.027	0.004	261.653	6.336	0.000
(Sub-Sahara Wintering) - Local Breeding	0.028	0.005	222.312	5.164	0.000
(Sub-Sahara Wintering) - Regional Breeding	0.029	0.004	189.364	6.739	0.000
(Sub-Sahara Wintering) - NW Africa Breeding	0.012	0.008	324.246	1.455	0.987
(Sub-Sahara Wintering) - (Sub-Sahara Breeding)	0.029	0.005	268.820	5.926	0.000
Local Spring - Regional Spring	0.000	0.004	264.214	-0.059	1.000
Local Spring - NW Africa Spring	-0.003	0.006	286.602	-0.589	1.000
Local Spring - (Sub-Sahara Spring)	-0.018	0.005	205.641	-3.440	0.055
Local Spring - Local Breeding	-0.017	0.004	266.342	-4.616	0.001
Local Spring - Regional Breeding	-0.015	0.004	270.145	-4.252	0.003
Local Spring - NW Africa Breeding	-0.033	0.008	323.119	-4.130	0.005
Local Spring - (Sub-Sahara Breeding)	-0.016	0.006	246.953	-2.762	0.299
Regional Spring - NW Africa Spring	-0.003	0.005	307.322	-0.623	1.000
Regional Spring - (Sub-Sahara Spring)	-0.017	0.004	197.008	-3.951	0.010

Table S9 *(continued)*

contrast	estimate	SE	df	t.ratio	p.value
Regional Spring - Local Breeding	-0.017	0.004	295.733	-4.189	0.004
Regional Spring - Regional Breeding	-0.015	0.002	264.058	-7.575	0.000
Regional Spring - NW Africa Breeding	-0.033	0.007	312.035	-4.388	0.002
Regional Spring - (Sub-Sahara Breeding)	-0.016	0.005	253.876	-3.060	0.152
NW Africa Spring - (Sub-Sahara Spring)	-0.014	0.006	262.244	-2.212	0.688
NW Africa Spring - Local Breeding	-0.014	0.006	296.931	-2.202	0.695
NW Africa Spring - Regional Breeding	-0.012	0.005	308.809	-2.267	0.648
NW Africa Spring - NW Africa Breeding	-0.029	0.008	276.861	-3.624	0.030
NW Africa Spring - (Sub-Sahara Breeding)	-0.012	0.007	283.067	-1.782	0.921
(Sub-Sahara Spring) - Local Breeding	0.001	0.005	230.080	0.110	1.000
(Sub-Sahara Spring) - Regional Breeding	0.002	0.004	201.688	0.505	1.000
(Sub-Sahara Spring) - NW Africa Breeding	-0.015	0.008	324.507	-1.811	0.911
(Sub-Sahara Spring) - (Sub-Sahara Breeding)	0.002	0.005	269.358	0.389	1.000
Local Breeding - Regional Breeding	0.002	0.004	298.938	0.406	1.000
Local Breeding - NW Africa Breeding	-0.016	0.008	322.520	-1.927	0.861
Local Breeding - (Sub-Sahara Breeding)	0.001	0.006	263.723	0.225	1.000
Regional Breeding - NW Africa Breeding	-0.017	0.007	311.683	-2.331	0.601
Regional Breeding - (Sub-Sahara Breeding)	0.000	0.005	256.677	-0.060	1.000
NW Africa Breeding - (Sub-Sahara Breeding)	0.017	0.009	325.060	1.961	0.845

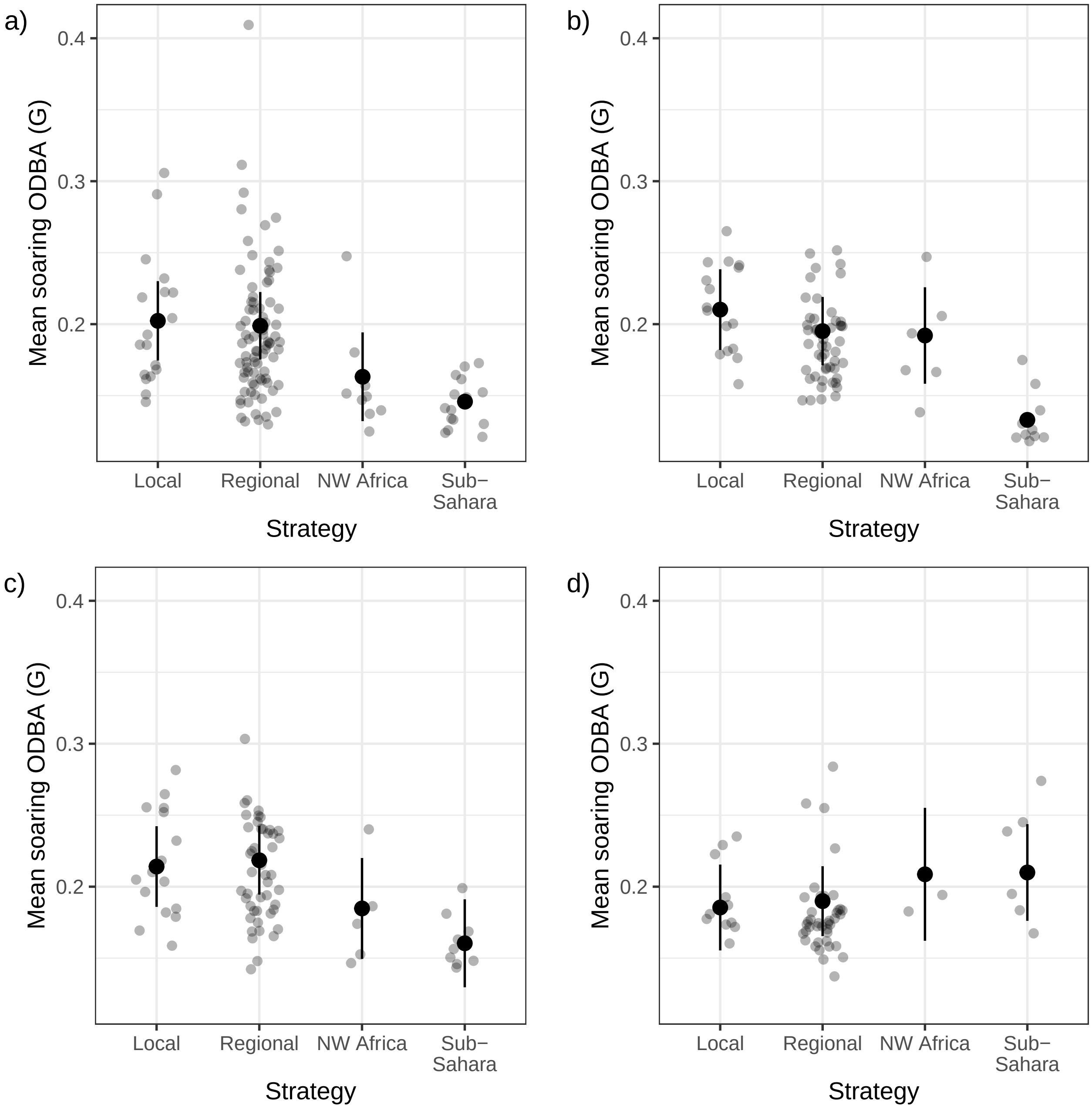

Fig S4: Relationship between the migratory strategy and the seasonal mean soaring ODBA, during the (a) autumn transition, (b) the wintering period, (c) the spring transition, and (d) the breeding period. Black dots are predicted estimates from the LMM, vertical lines are the confidence intervals based on fixed-effect uncertainty, and grey dots are raw data.

Table S10: Tukey's contrasts multiple comparisons with adjusted p-values for the seasonal mean soaring ODBA per migratory strategy and season.

contrast	estimate	SE	df	t.ratio	p.value
Local Autumn - Regional Autumn	0.003	0.009	233.704	0.388	1.000
Local Autumn - NW Africa Autumn	0.039	0.014	232.590	2.898	0.224
Local Autumn - (Sub-Sahara Autumn)	0.057	0.012	182.415	4.671	0.001
Local Autumn - Local Wintering	-0.008	0.009	266.823	-0.842	1.000
Local Autumn - Regional Wintering	0.007	0.009	242.891	0.767	1.000

Table S10 *(continued)*

contrast	estimate	SE	df	t.ratio	p.value
Local Autumn - NW Africa Wintering	0.010	0.015	270.183	0.679	1.000
Local Autumn - (Sub-Sahara Wintering)	0.069	0.013	209.358	5.207	0.000
Local Autumn - Local Spring	-0.012	0.009	266.823	-1.261	0.997
Local Autumn - Regional Spring	-0.016	0.009	243.788	-1.718	0.940
Local Autumn - NW Africa Spring	0.018	0.016	287.218	1.095	0.999
Local Autumn - (Sub-Sahara Spring)	0.042	0.014	220.573	3.084	0.145
Local Autumn - Local Breeding	0.017	0.011	272.057	1.604	0.966
Local Autumn - Regional Breeding	0.012	0.010	251.007	1.300	0.996
Local Autumn - NW Africa Breeding	-0.006	0.022	322.870	-0.284	1.000
Local Autumn - (Sub-Sahara Breeding)	-0.008	0.015	261.095	-0.498	1.000
Regional Autumn - NW Africa Autumn	0.036	0.011	246.325	3.123	0.130
Regional Autumn - (Sub-Sahara Autumn)	0.053	0.010	162.006	5.444	0.000
Regional Autumn - Local Wintering	-0.011	0.009	249.999	-1.221	0.998
Regional Autumn - Regional Wintering	0.004	0.005	269.053	0.762	1.000
Regional Autumn - NW Africa Wintering	0.007	0.013	290.820	0.513	1.000
Regional Autumn - (Sub-Sahara Wintering)	0.066	0.011	203.777	5.867	0.000
Regional Autumn - Local Spring	-0.015	0.009	249.999	-1.644	0.959
Regional Autumn - Regional Spring	-0.020	0.005	269.980	-3.948	0.010
Regional Autumn - NW Africa Spring	0.014	0.014	306.884	0.987	1.000
Regional Autumn - (Sub-Sahara Spring)	0.038	0.012	219.669	3.326	0.076
Regional Autumn - Local Breeding	0.013	0.011	286.967	1.261	0.997
Regional Autumn - Regional Breeding	0.009	0.005	269.535	1.706	0.944
Regional Autumn - NW Africa Breeding	-0.010	0.021	314.173	-0.466	1.000
Regional Autumn - (Sub-Sahara Breeding)	-0.011	0.014	273.786	-0.815	1.000
NW Africa Autumn - (Sub-Sahara Autumn)	0.017	0.014	209.663	1.243	0.997
NW Africa Autumn - Local Wintering	-0.047	0.014	239.244	-3.419	0.057
NW Africa Autumn - Regional Wintering	-0.032	0.012	257.257	-2.726	0.320
NW Africa Autumn - NW Africa Wintering	-0.029	0.015	281.325	-1.990	0.829
NW Africa Autumn - (Sub-Sahara Wintering)	0.030	0.015	228.295	2.004	0.821
NW Africa Autumn - Local Spring	-0.051	0.014	239.244	-3.703	0.023
NW Africa Autumn - Regional Spring	-0.055	0.012	257.785	-4.709	0.000
NW Africa Autumn - NW Africa Spring	-0.022	0.015	285.355	-1.397	0.991
NW Africa Autumn - (Sub-Sahara Spring)	0.003	0.015	236.806	0.182	1.000
NW Africa Autumn - Local Breeding	-0.022	0.015	260.268	-1.512	0.980
NW Africa Autumn - Regional Breeding	-0.027	0.012	262.097	-2.249	0.661
NW Africa Autumn - NW Africa Breeding	-0.045	0.022	284.161	-2.076	0.779
NW Africa Autumn - (Sub-Sahara Breeding)	-0.047	0.017	267.588	-2.775	0.290
(Sub-Sahara Autumn) - Local Wintering	-0.064	0.012	191.543	-5.205	0.000
(Sub-Sahara Autumn) - Regional Wintering	-0.049	0.010	176.068	-4.888	0.000
(Sub-Sahara Autumn) - NW Africa Wintering	-0.046	0.016	248.794	-2.972	0.189
(Sub-Sahara Autumn) - (Sub-Sahara Wintering)	0.013	0.011	280.266	1.134	0.999
(Sub-Sahara Autumn) - Local Spring	-0.068	0.012	191.543	-5.521	0.000
(Sub-Sahara Autumn) - Regional Spring	-0.073	0.010	176.838	-7.181	0.000
(Sub-Sahara Autumn) - NW Africa Spring	-0.039	0.016	268.366	-2.371	0.571
(Sub-Sahara Autumn) - (Sub-Sahara Spring)	-0.015	0.012	279.716	-1.260	0.997
(Sub-Sahara Autumn) - Local Breeding	-0.040	0.013	222.641	-2.951	0.199
(Sub-Sahara Autumn) - Regional Breeding	-0.044	0.010	183.682	-4.285	0.003
(Sub-Sahara Autumn) - NW Africa Breeding	-0.063	0.023	324.608	-2.782	0.285

Table S10 *(continued)*

contrast	estimate	SE	df	t.ratio	p.value
(Sub-Sahara Autumn) - (Sub-Sahara Breeding)	-0.064	0.013	281.392	-4.769	0.000
Local Wintering - Regional Wintering	0.015	0.010	257.078	1.547	0.976
Local Wintering - NW Africa Wintering	0.018	0.015	274.364	1.181	0.998
Local Wintering - (Sub-Sahara Wintering)	0.077	0.014	216.630	5.693	0.000
Local Wintering - Local Spring	-0.004	0.010	261.339	-0.410	1.000
Local Wintering - Regional Spring	-0.008	0.010	257.793	-0.852	1.000
Local Wintering - NW Africa Spring	0.025	0.016	290.154	1.565	0.973
Local Wintering - (Sub-Sahara Spring)	0.050	0.014	227.273	3.598	0.033
Local Wintering - Local Breeding	0.025	0.011	268.824	2.308	0.618
Local Wintering - Regional Breeding	0.020	0.010	263.988	2.048	0.796
Local Wintering - NW Africa Breeding	0.002	0.022	322.730	0.068	1.000
Local Wintering - (Sub-Sahara Breeding)	0.000	0.016	265.087	0.015	1.000
Regional Wintering - NW Africa Wintering	0.003	0.014	296.428	0.227	1.000
Regional Wintering - (Sub-Sahara Wintering)	0.062	0.012	214.559	5.388	0.000
Regional Wintering - Local Spring	-0.019	0.010	257.078	-1.950	0.850
Regional Wintering - Regional Spring	-0.023	0.005	263.168	-4.271	0.003
Regional Wintering - NW Africa Spring	0.010	0.015	310.385	0.714	1.000
Regional Wintering - (Sub-Sahara Spring)	0.035	0.012	229.415	2.929	0.209
Regional Wintering - Local Breeding	0.010	0.011	289.238	0.875	1.000
Regional Wintering - Regional Breeding	0.005	0.006	266.773	0.907	1.000
Regional Wintering - NW Africa Breeding	-0.014	0.021	313.641	-0.640	1.000
Regional Wintering - (Sub-Sahara Breeding)	-0.015	0.014	279.404	-1.070	1.000
NW Africa Wintering - (Sub-Sahara Wintering)	0.059	0.017	260.206	3.573	0.035
NW Africa Wintering - Local Spring	-0.022	0.015	274.364	-1.436	0.988
NW Africa Wintering - Regional Spring	-0.026	0.014	296.613	-1.954	0.848
NW Africa Wintering - NW Africa Spring	0.007	0.017	270.016	0.441	1.000
NW Africa Wintering - (Sub-Sahara Spring)	0.032	0.017	266.393	1.889	0.879
NW Africa Wintering - Local Breeding	0.007	0.016	286.817	0.408	1.000
NW Africa Wintering - Regional Breeding	0.002	0.014	298.591	0.160	1.000
NW Africa Wintering - NW Africa Breeding	-0.017	0.023	284.229	-0.725	1.000
NW Africa Wintering - (Sub-Sahara Breeding)	-0.018	0.018	287.199	-0.983	1.000
(Sub-Sahara Wintering) - Local Spring	-0.081	0.014	216.630	-5.982	0.000
(Sub-Sahara Wintering) - Regional Spring	-0.085	0.012	215.027	-7.399	0.000
(Sub-Sahara Wintering) - NW Africa Spring	-0.052	0.017	276.283	-2.983	0.183
(Sub-Sahara Wintering) - (Sub-Sahara Spring)	-0.027	0.012	262.614	-2.202	0.695
(Sub-Sahara Wintering) - Local Breeding	-0.052	0.015	241.023	-3.606	0.032
(Sub-Sahara Wintering) - Regional Breeding	-0.057	0.012	219.892	-4.861	0.000
(Sub-Sahara Wintering) - NW Africa Breeding	-0.076	0.023	324.467	-3.250	0.091
(Sub-Sahara Wintering) - (Sub-Sahara Breeding)	-0.077	0.014	271.935	-5.413	0.000
Local Spring - Regional Spring	-0.004	0.010	257.793	-0.451	1.000
Local Spring - NW Africa Spring	0.029	0.016	290.154	1.807	0.912
Local Spring - (Sub-Sahara Spring)	0.054	0.014	227.273	3.881	0.013
Local Spring - Local Breeding	0.029	0.011	268.824	2.673	0.354
Local Spring - Regional Breeding	0.024	0.010	263.988	2.443	0.517
Local Spring - NW Africa Breeding	0.005	0.022	322.730	0.242	1.000
Local Spring - (Sub-Sahara Breeding)	0.004	0.016	265.087	0.267	1.000
Regional Spring - NW Africa Spring	0.034	0.015	310.441	2.321	0.608
Regional Spring - (Sub-Sahara Spring)	0.058	0.012	229.780	4.888	0.000

Table S10 *(continued)*

contrast	estimate	SE	df	t.ratio	p.value
Regional Spring - Local Breeding	0.033	0.011	289.503	2.978	0.185
Regional Spring - Regional Breeding	0.029	0.006	265.686	4.912	0.000
Regional Spring - NW Africa Breeding	0.010	0.021	313.567	0.465	1.000
Regional Spring - (Sub-Sahara Breeding)	0.009	0.014	279.452	0.616	1.000
NW Africa Spring - (Sub-Sahara Spring)	0.024	0.018	281.157	1.387	0.992
NW Africa Spring - Local Breeding	-0.001	0.017	298.757	-0.039	1.000
NW Africa Spring - Regional Breeding	-0.005	0.015	311.541	-0.349	1.000
NW Africa Spring - NW Africa Breeding	-0.024	0.023	281.197	-1.023	1.000
NW Africa Spring - (Sub-Sahara Breeding)	-0.025	0.019	296.770	-1.329	0.995
(Sub-Sahara Spring) - Local Breeding	-0.025	0.015	249.614	-1.692	0.947
(Sub-Sahara Spring) - Regional Breeding	-0.029	0.012	234.156	-2.452	0.511
(Sub-Sahara Spring) - NW Africa Breeding	-0.048	0.023	324.503	-2.059	0.789
(Sub-Sahara Spring) - (Sub-Sahara Breeding)	-0.050	0.015	272.554	-3.416	0.057
Local Breeding - Regional Breeding	-0.004	0.011	292.830	-0.395	1.000
Local Breeding - NW Africa Breeding	-0.023	0.023	322.425	-1.007	1.000
Local Breeding - (Sub-Sahara Breeding)	-0.024	0.016	278.541	-1.496	0.982
Regional Breeding - NW Africa Breeding	-0.019	0.021	313.229	-0.885	1.000
Regional Breeding - (Sub-Sahara Breeding)	-0.020	0.014	281.388	-1.434	0.988
NW Africa Breeding - (Sub-Sahara Breeding)	-0.001	0.024	324.239	-0.052	1.000

Table S11: Effects for the GLMM for the foraging time per migratory strategy and season.

	Chisq	Df	Pr(>Chisq)
wintering_area	0.557	3	0.906
Stage	444.119	3	0.000
wintering_area:Stage	140.483	9	0.000

Table S12: Effects for the GLMM for the resting time per migratory strategy and season.

	Chisq	Df	Pr(>Chisq)
wintering_area	161.176	3	0
Stage	253.565	3	0
wintering_area:Stage	275.879	9	0

Table S13: Effects for the GLMM for the soaring time per migratory strategy and season.

	Chisq	Df	Pr(>Chisq)
wintering_area	455.851	3	0
Stage	67.554	3	0
wintering_area:Stage	444.775	9	0

Table S14: Effects for the GLMM for the flapping time per migratory strategy and season.

	Chisq	Df	Pr(>Chisq)
wintering_area	19.049	3	0

Table S14: Effects for the GLMM for the flapping time per migratory strategy and season. *(continued)*

	Chisq	Df	Pr(>Chisq)
Stage	429.825	3	0
wintering_area:Stage	55.433	9	0

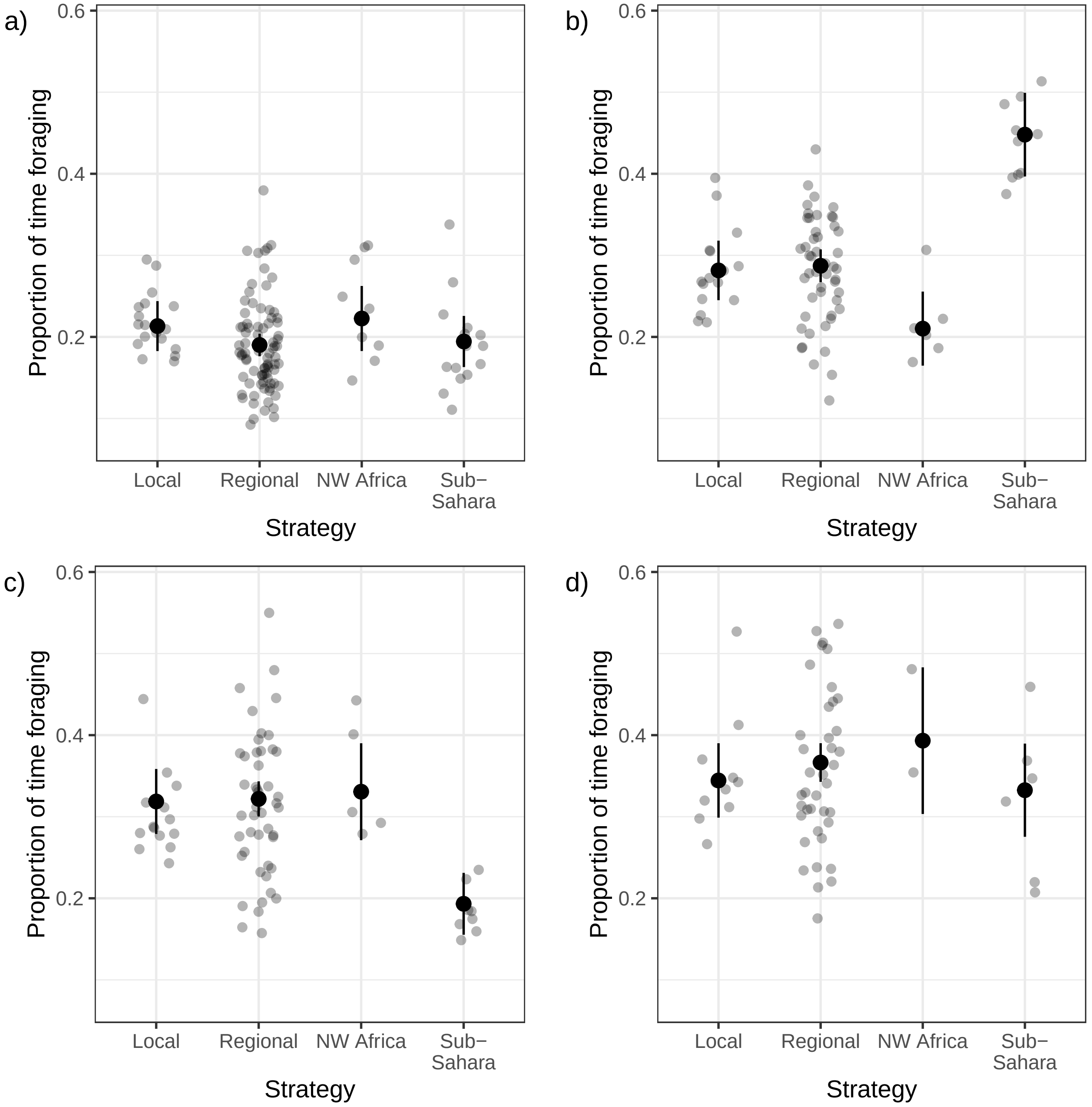

Fig S5: Relationship between the migratory strategy and foraging time, during the (a) autumn transition, (b) the wintering period, (c) the spring transition, and (d) the breeding period. Black dots are predicted estimates from the GLMM, vertical lines are the confidence intervals based on fixed-effect uncertainty, and grey dots are raw data.

Table S15: Tukey's contrasts multiple comparisons with adjusted p-values for the foraging time per migratory strategy and season.

contrast	estimate	SE	df	t.ratio	p.value
Local Autumn - Regional Autumn	0.144	0.101	321	1.425	0.989
Local Autumn - NW Africa Autumn	-0.055	0.149	321	-0.367	1.000
Local Autumn - (Sub-Sahara Autumn)	0.116	0.138	321	0.843	1.000
Local Autumn - Local Wintering	-0.368	0.095	321	-3.878	0.012
Local Autumn - Regional Wintering	-0.396	0.104	321	-3.808	0.015
Local Autumn - NW Africa Wintering	0.019	0.167	321	0.112	1.000
Local Autumn - (Sub-Sahara Wintering)	-1.096	0.141	321	-7.769	0.000
Local Autumn - Local Spring	-0.545	0.095	321	-5.758	0.000
Local Autumn - Regional Spring	-0.560	0.104	321	-5.369	0.000
Local Autumn - NW Africa Spring	-0.600	0.164	321	-3.648	0.027
Local Autumn - (Sub-Sahara Spring)	0.124	0.155	321	0.799	1.000
Local Autumn - Local Breeding	-0.662	0.103	321	-6.442	0.000
Local Autumn - Regional Breeding	-0.758	0.105	321	-7.210	0.000
Local Autumn - NW Africa Breeding	-0.871	0.212	321	-4.101	0.005
Local Autumn - (Sub-Sahara Breeding)	-0.608	0.161	321	-3.771	0.018
Regional Autumn - NW Africa Autumn	-0.198	0.125	321	-1.593	0.969
Regional Autumn - (Sub-Sahara Autumn)	-0.027	0.112	321	-0.245	1.000
Regional Autumn - Local Wintering	-0.512	0.100	321	-5.135	0.000
Regional Autumn - Regional Wintering	-0.540	0.051	321	-10.639	0.000
Regional Autumn - NW Africa Wintering	-0.125	0.145	321	-0.862	1.000
Regional Autumn - (Sub-Sahara Wintering)	-1.240	0.115	321	-10.771	0.000
Regional Autumn - Local Spring	-0.689	0.101	321	-6.793	0.000
Regional Autumn - Regional Spring	-0.704	0.051	321	-13.817	0.000
Regional Autumn - NW Africa Spring	-0.744	0.143	321	-5.215	0.000
Regional Autumn - (Sub-Sahara Spring)	-0.020	0.132	321	-0.151	1.000
Regional Autumn - Local Breeding	-0.806	0.110	321	-7.318	0.000
Regional Autumn - Regional Breeding	-0.901	0.052	321	-17.238	0.000
Regional Autumn - NW Africa Breeding	-1.015	0.195	321	-5.193	0.000
Regional Autumn - (Sub-Sahara Breeding)	-0.752	0.139	321	-5.414	0.000
NW Africa Autumn - (Sub-Sahara Autumn)	0.171	0.156	321	1.099	0.999
NW Africa Autumn - Local Wintering	-0.314	0.148	321	-2.116	0.754
NW Africa Autumn - Regional Wintering	-0.342	0.126	321	-2.706	0.331
NW Africa Autumn - NW Africa Wintering	0.073	0.152	321	0.483	1.000
NW Africa Autumn - (Sub-Sahara Wintering)	-1.042	0.158	321	-6.579	0.000
NW Africa Autumn - Local Spring	-0.491	0.149	321	-3.287	0.082
NW Africa Autumn - Regional Spring	-0.505	0.126	321	-3.999	0.008
NW Africa Autumn - NW Africa Spring	-0.545	0.150	321	-3.640	0.027
NW Africa Autumn - (Sub-Sahara Spring)	0.179	0.171	321	1.044	1.000
NW Africa Autumn - Local Breeding	-0.607	0.155	321	-3.911	0.011
NW Africa Autumn - Regional Breeding	-0.703	0.127	321	-5.536	0.000
NW Africa Autumn - NW Africa Breeding	-0.817	0.203	321	-4.024	0.007
NW Africa Autumn - (Sub-Sahara Breeding)	-0.554	0.177	321	-3.136	0.124
(Sub-Sahara Autumn) - Local Wintering	-0.485	0.137	321	-3.527	0.040
(Sub-Sahara Autumn) - Regional Wintering	-0.513	0.114	321	-4.511	0.001
(Sub-Sahara Autumn) - NW Africa Wintering	-0.098	0.173	321	-0.566	1.000
(Sub-Sahara Autumn) - (Sub-Sahara Wintering)	-1.213	0.111	321	-10.920	0.000
(Sub-Sahara Autumn) - Local Spring	-0.662	0.139	321	-4.778	0.000
(Sub-Sahara Autumn) - Regional Spring	-0.676	0.114	321	-5.945	0.000

Table S15 *(continued)*

contrast	estimate	SE	df	t.ratio	p.value
(Sub-Sahara Autumn) - NW Africa Spring	-0.716	0.171	321	-4.194	0.004
(Sub-Sahara Autumn) - (Sub-Sahara Spring)	0.007	0.129	321	0.058	1.000
(Sub-Sahara Autumn) - Local Breeding	-0.778	0.145	321	-5.362	0.000
(Sub-Sahara Autumn) - Regional Breeding	-0.874	0.115	321	-7.630	0.000
(Sub-Sahara Autumn) - NW Africa Breeding	-0.988	0.218	321	-4.539	0.001
(Sub-Sahara Autumn) - (Sub-Sahara Breeding)	-0.725	0.134	321	-5.420	0.000
Local Wintering - Regional Wintering	-0.028	0.103	321	-0.271	1.000
Local Wintering - NW Africa Wintering	0.387	0.166	321	2.328	0.603
Local Wintering - (Sub-Sahara Wintering)	-0.728	0.140	321	-5.185	0.000
Local Wintering - Local Spring	-0.177	0.093	321	-1.908	0.871
Local Wintering - Regional Spring	-0.192	0.103	321	-1.859	0.892
Local Wintering - NW Africa Spring	-0.232	0.164	321	-1.411	0.990
Local Wintering - (Sub-Sahara Spring)	0.492	0.155	321	3.185	0.109
Local Wintering - Local Breeding	-0.293	0.101	321	-2.896	0.223
Local Wintering - Regional Breeding	-0.389	0.104	321	-3.749	0.019
Local Wintering - NW Africa Breeding	-0.503	0.212	321	-2.371	0.571
Local Wintering - (Sub-Sahara Breeding)	-0.240	0.161	321	-1.494	0.983
Regional Wintering - NW Africa Wintering	0.415	0.147	321	2.831	0.257
Regional Wintering - (Sub-Sahara Wintering)	-0.700	0.117	321	-5.988	0.000
Regional Wintering - Local Spring	-0.149	0.105	321	-1.425	0.989
Regional Wintering - Regional Spring	-0.164	0.053	321	-3.060	0.151
Regional Wintering - NW Africa Spring	-0.204	0.144	321	-1.415	0.990
Regional Wintering - (Sub-Sahara Spring)	0.520	0.134	321	3.889	0.012
Regional Wintering - Local Breeding	-0.265	0.113	321	-2.346	0.590
Regional Wintering - Regional Breeding	-0.361	0.055	321	-6.560	0.000
Regional Wintering - NW Africa Breeding	-0.475	0.196	321	-2.419	0.535
Regional Wintering - (Sub-Sahara Breeding)	-0.212	0.140	321	-1.510	0.981
NW Africa Wintering - (Sub-Sahara Wintering)	-1.115	0.175	321	-6.363	0.000
NW Africa Wintering - Local Spring	-0.564	0.167	321	-3.375	0.064
NW Africa Wintering - Regional Spring	-0.579	0.147	321	-3.946	0.009
NW Africa Wintering - NW Africa Spring	-0.619	0.163	321	-3.794	0.016
NW Africa Wintering - (Sub-Sahara Spring)	0.105	0.187	321	0.564	1.000
NW Africa Wintering - Local Breeding	-0.680	0.172	321	-3.945	0.009
NW Africa Wintering - Regional Breeding	-0.776	0.147	321	-5.276	0.000
NW Africa Wintering - NW Africa Breeding	-0.890	0.215	321	-4.145	0.004
NW Africa Wintering - (Sub-Sahara Breeding)	-0.627	0.192	321	-3.268	0.087
(Sub-Sahara Wintering) - Local Spring	0.551	0.141	321	3.897	0.011
(Sub-Sahara Wintering) - Regional Spring	0.536	0.117	321	4.584	0.001
(Sub-Sahara Wintering) - NW Africa Spring	0.496	0.173	321	2.866	0.238
(Sub-Sahara Wintering) - (Sub-Sahara Spring)	1.220	0.126	321	9.667	0.000
(Sub-Sahara Wintering) - Local Breeding	0.435	0.148	321	2.939	0.202
(Sub-Sahara Wintering) - Regional Breeding	0.339	0.118	321	2.876	0.233
(Sub-Sahara Wintering) - NW Africa Breeding	0.225	0.220	321	1.025	1.000
(Sub-Sahara Wintering) - (Sub-Sahara Breeding)	0.488	0.132	321	3.710	0.022
Local Spring - Regional Spring	-0.015	0.105	321	-0.139	1.000
Local Spring - NW Africa Spring	-0.055	0.165	321	-0.331	1.000
Local Spring - (Sub-Sahara Spring)	0.669	0.155	321	4.305	0.002
Local Spring - Local Breeding	-0.116	0.101	321	-1.157	0.999

Table S15 *(continued)*

contrast	estimate	SE	df	t.ratio	p.value
Local Spring - Regional Breeding	-0.212	0.106	321	-2.009	0.818
Local Spring - NW Africa Breeding	-0.326	0.213	321	-1.532	0.978
Local Spring - (Sub-Sahara Breeding)	-0.063	0.162	321	-0.390	1.000
Regional Spring - NW Africa Spring	-0.040	0.144	321	-0.278	1.000
Regional Spring - (Sub-Sahara Spring)	0.684	0.134	321	5.109	0.000
Regional Spring - Local Breeding	-0.102	0.113	321	-0.898	1.000
Regional Spring - Regional Breeding	-0.198	0.055	321	-3.605	0.031
Regional Spring - NW Africa Breeding	-0.311	0.196	321	-1.586	0.970
Regional Spring - (Sub-Sahara Breeding)	-0.048	0.140	321	-0.344	1.000
NW Africa Spring - (Sub-Sahara Spring)	0.724	0.185	321	3.916	0.010
NW Africa Spring - Local Breeding	-0.062	0.170	321	-0.363	1.000
NW Africa Spring - Regional Breeding	-0.158	0.145	321	-1.091	0.999
NW Africa Spring - NW Africa Breeding	-0.271	0.212	321	-1.281	0.996
NW Africa Spring - (Sub-Sahara Breeding)	-0.008	0.190	321	-0.044	1.000
(Sub-Sahara Spring) - Local Breeding	-0.786	0.161	321	-4.868	0.000
(Sub-Sahara Spring) - Regional Breeding	-0.882	0.135	321	-6.553	0.000
(Sub-Sahara Spring) - NW Africa Breeding	-0.995	0.229	321	-4.350	0.002
(Sub-Sahara Spring) - (Sub-Sahara Breeding)	-0.732	0.148	321	-4.954	0.000
Local Breeding - Regional Breeding	-0.096	0.114	321	-0.841	1.000
Local Breeding - NW Africa Breeding	-0.210	0.217	321	-0.966	1.000
Local Breeding - (Sub-Sahara Breeding)	0.053	0.167	321	0.319	1.000
Regional Breeding - NW Africa Breeding	-0.114	0.197	321	-0.578	1.000
Regional Breeding - (Sub-Sahara Breeding)	0.149	0.141	321	1.058	1.000
NW Africa Breeding - (Sub-Sahara Breeding)	0.263	0.233	321	1.129	0.999

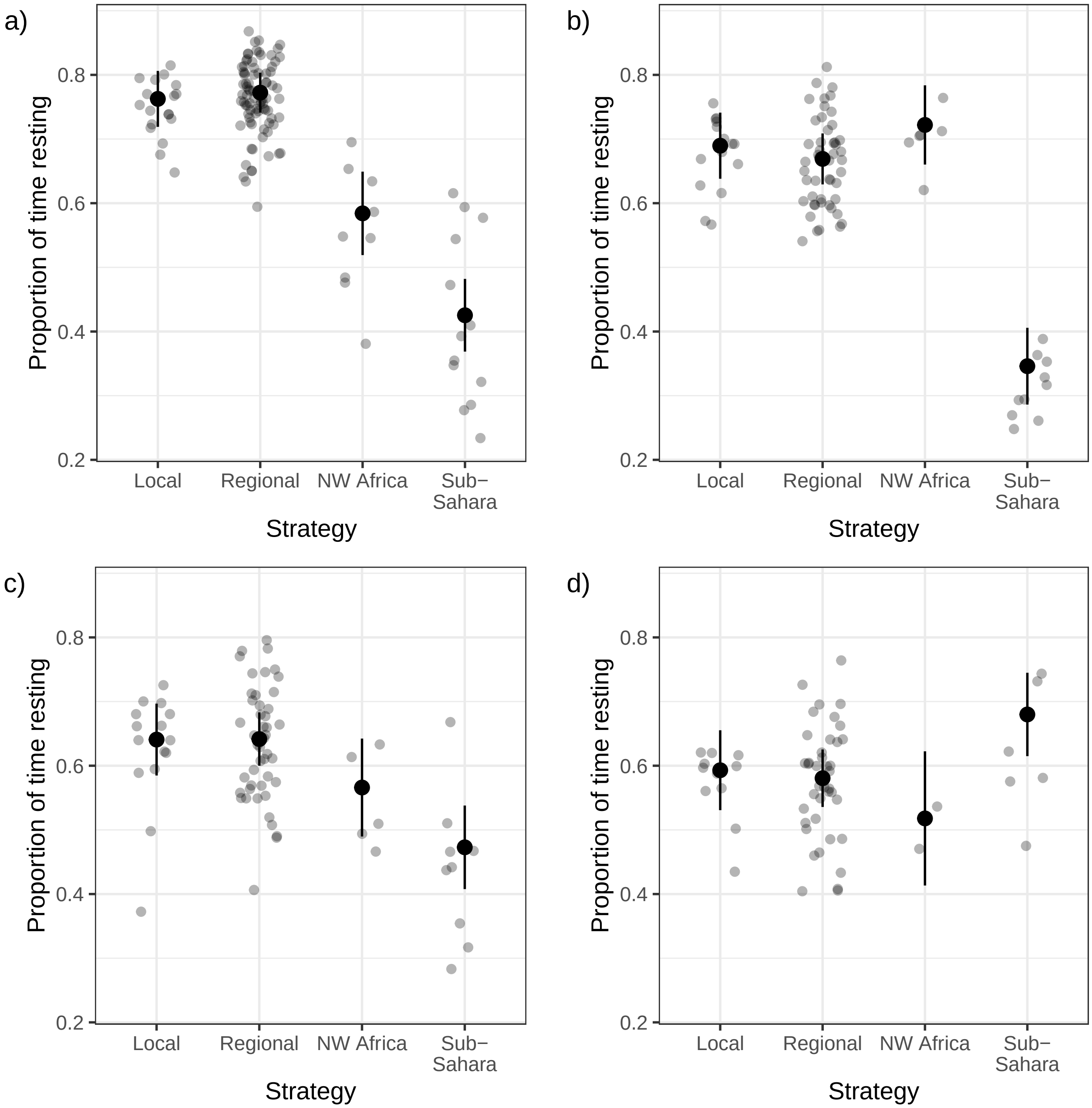

Fig S6: Relationship between the migratory strategy and resting time, during the (a) autumn transition, (b) the wintering period, (c) the spring transition, and (d) the breeding period. Black dots are predicted estimates from the GLMM, vertical lines are the confidence intervals based on fixed-effect uncertainty, and grey dots are raw data.

Table S16: Tukey's contrasts multiple comparisons with adjusted p-values for the resting time per migratory strategy and season.

contrast	estimate	SE	df	t.ratio	p.value
Local Autumn - Regional Autumn	-0.055	0.098	321	-0.555	1.000
Local Autumn - NW Africa Autumn	0.827	0.140	321	5.903	0.000
Local Autumn - (Sub-Sahara Autumn)	1.468	0.129	321	11.356	0.000
Local Autumn - Local Wintering	0.369	0.096	321	3.843	0.014
Local Autumn - Regional Wintering	0.463	0.102	321	4.539	0.001

Table S16 *(continued)*

contrast	estimate	SE	df	t.ratio	p.value
Local Autumn - NW Africa Wintering	0.213	0.160	321	1.327	0.995
Local Autumn - (Sub-Sahara Wintering)	1.804	0.144	321	12.527	0.000
Local Autumn - Local Spring	0.588	0.096	321	6.141	0.000
Local Autumn - Regional Spring	0.585	0.102	321	5.716	0.000
Local Autumn - NW Africa Spring	0.901	0.162	321	5.572	0.000
Local Autumn - (Sub-Sahara Spring)	1.276	0.142	321	8.961	0.000
Local Autumn - Local Breeding	0.791	0.104	321	7.573	0.000
Local Autumn - Regional Breeding	0.842	0.103	321	8.166	0.000
Local Autumn - NW Africa Breeding	1.095	0.216	321	5.061	0.000
Local Autumn - (Sub-Sahara Breeding)	0.414	0.159	321	2.600	0.402
Regional Autumn - NW Africa Autumn	0.882	0.116	321	7.610	0.000
Regional Autumn - (Sub-Sahara Autumn)	1.522	0.102	321	14.907	0.000
Regional Autumn - Local Wintering	0.423	0.098	321	4.314	0.002
Regional Autumn - Regional Wintering	0.518	0.050	321	10.271	0.000
Regional Autumn - NW Africa Wintering	0.267	0.139	321	1.923	0.864
Regional Autumn - (Sub-Sahara Wintering)	1.858	0.121	321	15.390	0.000
Regional Autumn - Local Spring	0.643	0.100	321	6.434	0.000
Regional Autumn - Regional Spring	0.639	0.051	321	12.559	0.000
Regional Autumn - NW Africa Spring	0.956	0.141	321	6.799	0.000
Regional Autumn - (Sub-Sahara Spring)	1.330	0.119	321	11.186	0.000
Regional Autumn - Local Breeding	0.845	0.109	321	7.750	0.000
Regional Autumn - Regional Breeding	0.896	0.052	321	17.088	0.000
Regional Autumn - NW Africa Breeding	1.150	0.200	321	5.745	0.000
Regional Autumn - (Sub-Sahara Breeding)	0.468	0.139	321	3.360	0.066
NW Africa Autumn - (Sub-Sahara Autumn)	0.641	0.142	321	4.504	0.001
NW Africa Autumn - Local Wintering	-0.458	0.140	321	-3.270	0.086
NW Africa Autumn - Regional Wintering	-0.364	0.118	321	-3.083	0.142
NW Africa Autumn - NW Africa Wintering	-0.614	0.143	321	-4.293	0.002
NW Africa Autumn - (Sub-Sahara Wintering)	0.977	0.156	321	6.274	0.000
NW Africa Autumn - Local Spring	-0.239	0.141	321	-1.690	0.948
NW Africa Autumn - Regional Spring	-0.242	0.118	321	-2.050	0.795
NW Africa Autumn - NW Africa Spring	0.074	0.145	321	0.512	1.000
NW Africa Autumn - (Sub-Sahara Spring)	0.449	0.154	321	2.910	0.216
NW Africa Autumn - Local Breeding	-0.036	0.148	321	-0.247	1.000
NW Africa Autumn - Regional Breeding	0.015	0.119	321	0.125	1.000
NW Africa Autumn - NW Africa Breeding	0.268	0.205	321	1.307	0.995
NW Africa Autumn - (Sub-Sahara Breeding)	-0.413	0.170	321	-2.437	0.521
(Sub-Sahara Autumn) - Local Wintering	-1.099	0.129	321	-8.502	0.000
(Sub-Sahara Autumn) - Regional Wintering	-1.005	0.104	321	-9.619	0.000
(Sub-Sahara Autumn) - NW Africa Wintering	-1.255	0.162	321	-7.745	0.000
(Sub-Sahara Autumn) - (Sub-Sahara Wintering)	0.336	0.112	321	3.010	0.171
(Sub-Sahara Autumn) - Local Spring	-0.880	0.130	321	-6.742	0.000
(Sub-Sahara Autumn) - Regional Spring	-0.883	0.105	321	-8.433	0.000
(Sub-Sahara Autumn) - NW Africa Spring	-0.566	0.164	321	-3.462	0.049
(Sub-Sahara Autumn) - (Sub-Sahara Spring)	-0.192	0.110	321	-1.752	0.931
(Sub-Sahara Autumn) - Local Breeding	-0.677	0.138	321	-4.921	0.000
(Sub-Sahara Autumn) - Regional Breeding	-0.626	0.106	321	-5.917	0.000
(Sub-Sahara Autumn) - NW Africa Breeding	-0.372	0.218	321	-1.709	0.943

Table S16 *(continued)*

contrast	estimate	SE	df	t.ratio	p.value
(Sub-Sahara Autumn) - (Sub-Sahara Breeding)	-1.054	0.130	321	-8.124	0.000
Local Wintering - Regional Wintering	0.095	0.102	321	0.929	1.000
Local Wintering - NW Africa Wintering	-0.156	0.160	321	-0.973	1.000
Local Wintering - (Sub-Sahara Wintering)	1.435	0.144	321	9.954	0.000
Local Wintering - Local Spring	0.220	0.095	321	2.324	0.607
Local Wintering - Regional Spring	0.216	0.102	321	2.118	0.752
Local Wintering - NW Africa Spring	0.533	0.162	321	3.293	0.081
Local Wintering - (Sub-Sahara Spring)	0.907	0.143	321	6.363	0.000
Local Wintering - Local Breeding	0.422	0.103	321	4.079	0.006
Local Wintering - Regional Breeding	0.473	0.103	321	4.604	0.001
Local Wintering - NW Africa Breeding	0.727	0.216	321	3.358	0.067
Local Wintering - (Sub-Sahara Breeding)	0.045	0.159	321	0.283	1.000
Regional Wintering - NW Africa Wintering	-0.251	0.141	321	-1.781	0.922
Regional Wintering - (Sub-Sahara Wintering)	1.341	0.123	321	10.919	0.000
Regional Wintering - Local Spring	0.125	0.104	321	1.206	0.998
Regional Wintering - Regional Spring	0.122	0.054	321	2.247	0.663
Regional Wintering - NW Africa Spring	0.438	0.142	321	3.079	0.144
Regional Wintering - (Sub-Sahara Spring)	0.812	0.121	321	6.715	0.000
Regional Wintering - Local Breeding	0.327	0.113	321	2.906	0.218
Regional Wintering - Regional Breeding	0.379	0.056	321	6.773	0.000
Regional Wintering - NW Africa Breeding	0.632	0.201	321	3.142	0.122
Regional Wintering - (Sub-Sahara Breeding)	-0.050	0.141	321	-0.351	1.000
NW Africa Wintering - (Sub-Sahara Wintering)	1.591	0.174	321	9.147	0.000
NW Africa Wintering - Local Spring	0.376	0.161	321	2.330	0.602
NW Africa Wintering - Regional Spring	0.372	0.141	321	2.641	0.374
NW Africa Wintering - NW Africa Spring	0.689	0.161	321	4.286	0.003
NW Africa Wintering - (Sub-Sahara Spring)	1.063	0.173	321	6.158	0.000
NW Africa Wintering - Local Breeding	0.578	0.167	321	3.459	0.049
NW Africa Wintering - Regional Breeding	0.629	0.141	321	4.450	0.001
NW Africa Wintering - NW Africa Breeding	0.883	0.219	321	4.025	0.007
NW Africa Wintering - (Sub-Sahara Breeding)	0.201	0.187	321	1.077	0.999
(Sub-Sahara Wintering) - Local Spring	-1.215	0.145	321	-8.371	0.000
(Sub-Sahara Wintering) - Regional Spring	-1.219	0.123	321	-9.906	0.000
(Sub-Sahara Wintering) - NW Africa Spring	-0.902	0.176	321	-5.139	0.000
(Sub-Sahara Wintering) - (Sub-Sahara Spring)	-0.528	0.121	321	-4.357	0.002
(Sub-Sahara Wintering) - Local Breeding	-1.013	0.152	321	-6.686	0.000
(Sub-Sahara Wintering) - Regional Breeding	-0.962	0.124	321	-7.757	0.000
(Sub-Sahara Wintering) - NW Africa Breeding	-0.708	0.227	321	-3.119	0.130
(Sub-Sahara Wintering) - (Sub-Sahara Breeding)	-1.390	0.140	321	-9.928	0.000
Local Spring - Regional Spring	-0.004	0.104	321	-0.034	1.000
Local Spring - NW Africa Spring	0.313	0.163	321	1.924	0.863
Local Spring - (Sub-Sahara Spring)	0.687	0.144	321	4.787	0.000
Local Spring - Local Breeding	0.202	0.103	321	1.967	0.841
Local Spring - Regional Breeding	0.254	0.105	321	2.421	0.533
Local Spring - NW Africa Breeding	0.507	0.217	321	2.335	0.598
Local Spring - (Sub-Sahara Breeding)	-0.175	0.160	321	-1.090	0.999
Regional Spring - NW Africa Spring	0.317	0.142	321	2.223	0.680
Regional Spring - (Sub-Sahara Spring)	0.691	0.121	321	5.697	0.000

Table S16 *(continued)*

contrast	estimate	SE	df	t.ratio	p.value
Regional Spring - Local Breeding	0.206	0.113	321	1.823	0.906
Regional Spring - Regional Breeding	0.257	0.056	321	4.589	0.001
Regional Spring - NW Africa Breeding	0.511	0.201	321	2.538	0.447
Regional Spring - (Sub-Sahara Breeding)	-0.171	0.142	321	-1.209	0.998
NW Africa Spring - (Sub-Sahara Spring)	0.374	0.174	321	2.148	0.732
NW Africa Spring - Local Breeding	-0.111	0.169	321	-0.657	1.000
NW Africa Spring - Regional Breeding	-0.059	0.143	321	-0.416	1.000
NW Africa Spring - NW Africa Breeding	0.194	0.219	321	0.888	1.000
NW Africa Spring - (Sub-Sahara Breeding)	-0.488	0.188	321	-2.590	0.409
(Sub-Sahara Spring) - Local Breeding	-0.485	0.150	321	-3.234	0.095
(Sub-Sahara Spring) - Regional Breeding	-0.434	0.122	321	-3.549	0.037
(Sub-Sahara Spring) - NW Africa Breeding	-0.180	0.226	321	-0.797	1.000
(Sub-Sahara Spring) - (Sub-Sahara Breeding)	-0.862	0.138	321	-6.246	0.000
Local Breeding - Regional Breeding	0.051	0.114	321	0.451	1.000
Local Breeding - NW Africa Breeding	0.305	0.222	321	1.376	0.992
Local Breeding - (Sub-Sahara Breeding)	-0.377	0.166	321	-2.272	0.644
Regional Breeding - NW Africa Breeding	0.254	0.202	321	1.258	0.997
Regional Breeding - (Sub-Sahara Breeding)	-0.428	0.142	321	-3.009	0.171
NW Africa Breeding - (Sub-Sahara Breeding)	-0.682	0.237	321	-2.874	0.234

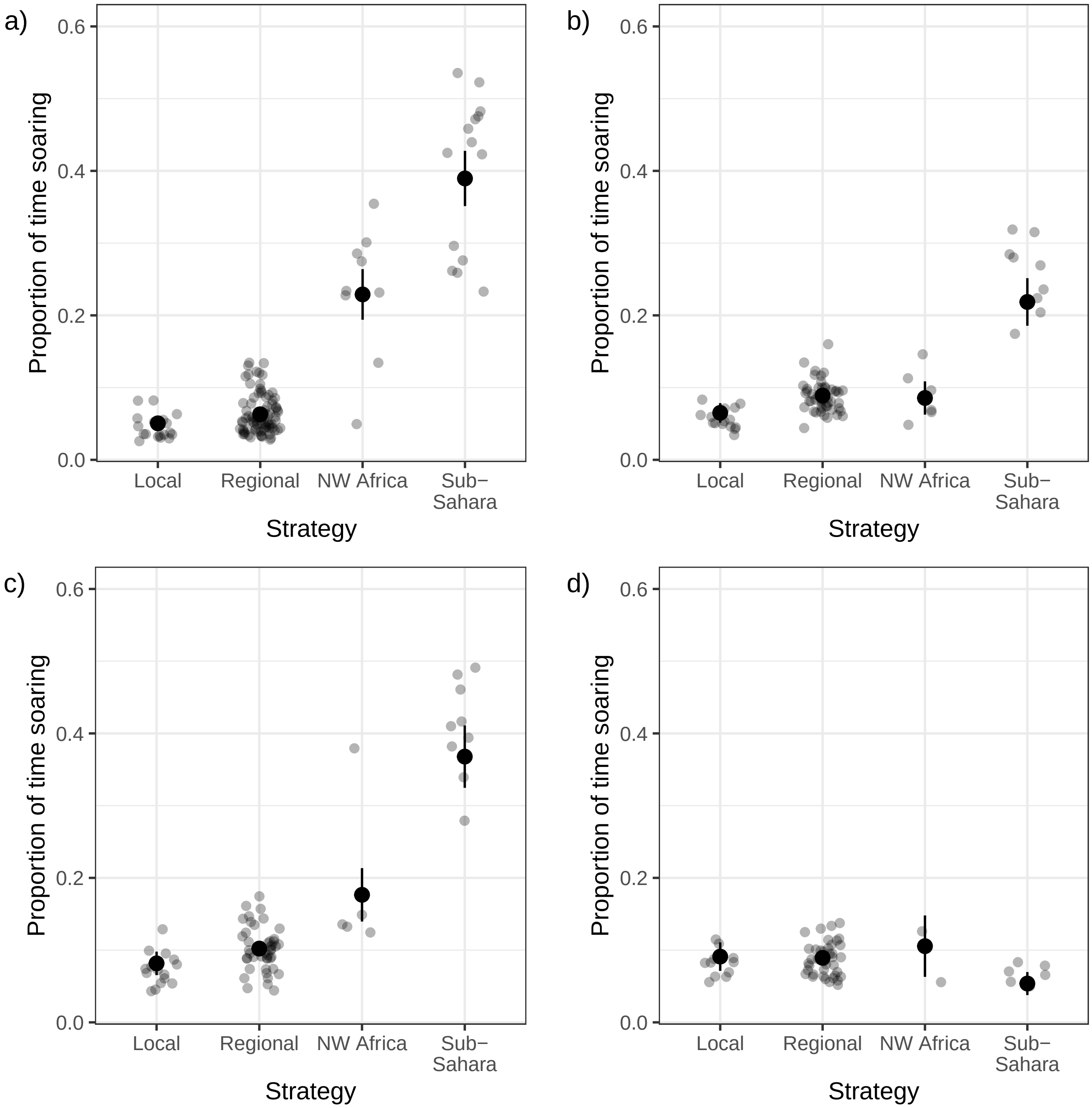

Fig S7: Relationship between the migratory strategy and soaring time, during the (a) autumn transition, (b) the wintering period, (c) the spring transition, and (d) the breeding period. Black dots are predicted estimates from the GLMM, vertical lines are the confidence intervals based on fixed-effect uncertainty, and grey dots are raw data.

Table S17: Tukey's contrasts multiple comparisons with adjusted p-values for the soaring time per migratory strategy and season.

contrast	estimate	SE	df	t.ratio	p.value
Local Autumn - Regional Autumn	-0.234	0.123	319	-1.902	0.873
Local Autumn - NW Africa Autumn	-1.720	0.153	319	-11.237	0.000
Local Autumn - (Sub-Sahara Autumn)	-2.484	0.141	319	-17.563	0.000
Local Autumn - Local Wintering	-0.268	0.132	319	-2.034	0.804
Local Autumn - Regional Wintering	-0.608	0.126	319	-4.819	0.000

Table S17 *(continued)*

contrast	estimate	SE	df	t.ratio	p.value
Local Autumn - NW Africa Wintering	-0.565	0.189	319	-2.983	0.182
Local Autumn - (Sub-Sahara Wintering)	-1.659	0.152	319	-10.916	0.000
Local Autumn - Local Spring	-0.513	0.128	319	-4.008	0.007
Local Autumn - Regional Spring	-0.760	0.126	319	-6.027	0.000
Local Autumn - NW Africa Spring	-1.394	0.173	319	-8.049	0.000
Local Autumn - (Sub-Sahara Spring)	-2.392	0.150	319	-15.989	0.000
Local Autumn - Local Breeding	-0.633	0.137	319	-4.610	0.001
Local Autumn - Regional Breeding	-0.612	0.128	319	-4.762	0.000
Local Autumn - NW Africa Breeding	-0.796	0.257	319	-3.102	0.136
Local Autumn - (Sub-Sahara Breeding)	-0.065	0.198	319	-0.329	1.000
Regional Autumn - NW Africa Autumn	-1.486	0.111	319	-13.372	0.000
Regional Autumn - (Sub-Sahara Autumn)	-2.250	0.096	319	-23.421	0.000
Regional Autumn - Local Wintering	-0.034	0.121	319	-0.277	1.000
Regional Autumn - Regional Wintering	-0.374	0.059	319	-6.318	0.000
Regional Autumn - NW Africa Wintering	-0.331	0.157	319	-2.110	0.758
Regional Autumn - (Sub-Sahara Wintering)	-1.426	0.110	319	-12.966	0.000
Regional Autumn - Local Spring	-0.279	0.119	319	-2.341	0.593
Regional Autumn - Regional Spring	-0.526	0.059	319	-8.883	0.000
Regional Autumn - NW Africa Spring	-1.160	0.137	319	-8.466	0.000
Regional Autumn - (Sub-Sahara Spring)	-2.158	0.107	319	-20.208	0.000
Regional Autumn - Local Breeding	-0.399	0.129	319	-3.086	0.141
Regional Autumn - Regional Breeding	-0.378	0.064	319	-5.939	0.000
Regional Autumn - NW Africa Breeding	-0.562	0.234	319	-2.405	0.545
Regional Autumn - (Sub-Sahara Breeding)	0.169	0.167	319	1.011	1.000
NW Africa Autumn - (Sub-Sahara Autumn)	-0.765	0.130	319	-5.879	0.000
NW Africa Autumn - Local Wintering	1.452	0.152	319	9.572	0.000
NW Africa Autumn - Regional Wintering	1.111	0.113	319	9.819	0.000
NW Africa Autumn - NW Africa Wintering	1.155	0.150	319	7.707	0.000
NW Africa Autumn - (Sub-Sahara Wintering)	0.060	0.141	319	0.427	1.000
NW Africa Autumn - Local Spring	1.206	0.150	319	8.037	0.000
NW Africa Autumn - Regional Spring	0.960	0.113	319	8.511	0.000
NW Africa Autumn - NW Africa Spring	0.326	0.132	319	2.475	0.493
NW Africa Autumn - (Sub-Sahara Spring)	-0.673	0.138	319	-4.869	0.000
NW Africa Autumn - Local Breeding	1.087	0.158	319	6.871	0.000
NW Africa Autumn - Regional Breeding	1.108	0.115	319	9.608	0.000
NW Africa Autumn - NW Africa Breeding	0.924	0.232	319	3.973	0.008
NW Africa Autumn - (Sub-Sahara Breeding)	1.655	0.189	319	8.765	0.000
(Sub-Sahara Autumn) - Local Wintering	2.217	0.140	319	15.831	0.000
(Sub-Sahara Autumn) - Regional Wintering	1.876	0.099	319	19.009	0.000
(Sub-Sahara Autumn) - NW Africa Wintering	1.919	0.171	319	11.231	0.000
(Sub-Sahara Autumn) - (Sub-Sahara Wintering)	0.825	0.083	319	9.954	0.000
(Sub-Sahara Autumn) - Local Spring	1.971	0.138	319	14.257	0.000
(Sub-Sahara Autumn) - Regional Spring	1.724	0.099	319	17.469	0.000
(Sub-Sahara Autumn) - NW Africa Spring	1.091	0.153	319	7.113	0.000
(Sub-Sahara Autumn) - (Sub-Sahara Spring)	0.092	0.079	319	1.161	0.999
(Sub-Sahara Autumn) - Local Breeding	1.852	0.147	319	12.596	0.000
(Sub-Sahara Autumn) - Regional Breeding	1.872	0.102	319	18.401	0.000
(Sub-Sahara Autumn) - NW Africa Breeding	1.688	0.244	319	6.912	0.000

Table S17 *(continued)*

contrast	estimate	SE	df	t.ratio	p.value
(Sub-Sahara Autumn) - (Sub-Sahara Breeding)	2.419	0.150	319	16.137	0.000
Local Wintering - Regional Wintering	-0.341	0.125	319	-2.736	0.313
Local Wintering - NW Africa Wintering	-0.297	0.188	319	-1.579	0.971
Local Wintering - (Sub-Sahara Wintering)	-1.392	0.151	319	-9.233	0.000
Local Wintering - Local Spring	-0.246	0.124	319	-1.982	0.834
Local Wintering - Regional Spring	-0.492	0.124	319	-3.958	0.009
Local Wintering - NW Africa Spring	-1.126	0.172	319	-6.549	0.000
Local Wintering - (Sub-Sahara Spring)	-2.125	0.148	319	-14.325	0.000
Local Wintering - Local Breeding	-0.365	0.133	319	-2.738	0.312
Local Wintering - Regional Breeding	-0.344	0.127	319	-2.715	0.326
Local Wintering - NW Africa Breeding	-0.528	0.256	319	-2.066	0.785
Local Wintering - (Sub-Sahara Breeding)	0.203	0.197	319	1.030	1.000
Regional Wintering - NW Africa Wintering	0.043	0.158	319	0.273	1.000
Regional Wintering - (Sub-Sahara Wintering)	-1.051	0.112	319	-9.368	0.000
Regional Wintering - Local Spring	0.095	0.123	319	0.772	1.000
Regional Wintering - Regional Spring	-0.152	0.062	319	-2.454	0.508
Regional Wintering - NW Africa Spring	-0.785	0.139	319	-5.666	0.000
Regional Wintering - (Sub-Sahara Spring)	-1.784	0.109	319	-16.345	0.000
Regional Wintering - Local Breeding	-0.024	0.133	319	-0.185	1.000
Regional Wintering - Regional Breeding	-0.004	0.067	319	-0.053	1.000
Regional Wintering - NW Africa Breeding	-0.188	0.235	319	-0.800	1.000
Regional Wintering - (Sub-Sahara Breeding)	0.543	0.169	319	3.221	0.099
NW Africa Wintering - (Sub-Sahara Wintering)	-1.095	0.179	319	-6.128	0.000
NW Africa Wintering - Local Spring	0.052	0.187	319	0.275	1.000
NW Africa Wintering - Regional Spring	-0.195	0.158	319	-1.233	0.998
NW Africa Wintering - NW Africa Spring	-0.829	0.169	319	-4.916	0.000
NW Africa Wintering - (Sub-Sahara Spring)	-1.827	0.177	319	-10.336	0.000
NW Africa Wintering - Local Breeding	-0.068	0.194	319	-0.350	1.000
NW Africa Wintering - Regional Breeding	-0.047	0.160	319	-0.293	1.000
NW Africa Wintering - NW Africa Breeding	-0.231	0.259	319	-0.892	1.000
NW Africa Wintering - (Sub-Sahara Breeding)	0.500	0.218	319	2.289	0.632
(Sub-Sahara Wintering) - Local Spring	1.146	0.149	319	7.682	0.000
(Sub-Sahara Wintering) - Regional Spring	0.900	0.112	319	8.011	0.000
(Sub-Sahara Wintering) - NW Africa Spring	0.266	0.162	319	1.637	0.960
(Sub-Sahara Wintering) - (Sub-Sahara Spring)	-0.733	0.089	319	-8.239	0.000
(Sub-Sahara Wintering) - Local Breeding	1.027	0.157	319	6.530	0.000
(Sub-Sahara Wintering) - Regional Breeding	1.048	0.115	319	9.116	0.000
(Sub-Sahara Wintering) - NW Africa Breeding	0.864	0.250	319	3.448	0.051
(Sub-Sahara Wintering) - (Sub-Sahara Breeding)	1.595	0.154	319	10.329	0.000
Local Spring - Regional Spring	-0.247	0.123	319	-2.009	0.819
Local Spring - NW Africa Spring	-0.880	0.171	319	-5.162	0.000
Local Spring - (Sub-Sahara Spring)	-1.879	0.147	319	-12.806	0.000
Local Spring - Local Breeding	-0.119	0.129	319	-0.922	1.000
Local Spring - Regional Breeding	-0.098	0.125	319	-0.786	1.000
Local Spring - NW Africa Breeding	-0.282	0.255	319	-1.109	0.999
Local Spring - (Sub-Sahara Breeding)	0.448	0.196	319	2.293	0.629
Regional Spring - NW Africa Spring	-0.634	0.138	319	-4.588	0.001
Regional Spring - (Sub-Sahara Spring)	-1.632	0.109	319	-14.947	0.000

Table S17 *(continued)*

contrast	estimate	SE	df	t.ratio	p.value
Regional Spring - Local Breeding	0.127	0.132	319	0.961	1.000
Regional Spring - Regional Breeding	0.148	0.066	319	2.249	0.661
Regional Spring - NW Africa Breeding	-0.036	0.234	319	-0.154	1.000
Regional Spring - (Sub-Sahara Breeding)	0.695	0.169	319	4.119	0.005
NW Africa Spring - (Sub-Sahara Spring)	-0.999	0.160	319	-6.232	0.000
NW Africa Spring - Local Breeding	0.761	0.178	319	4.282	0.003
NW Africa Spring - Regional Breeding	0.782	0.140	319	5.578	0.000
NW Africa Spring - NW Africa Breeding	0.598	0.244	319	2.446	0.514
NW Africa Spring - (Sub-Sahara Breeding)	1.329	0.205	319	6.467	0.000
(Sub-Sahara Spring) - Local Breeding	1.760	0.155	319	11.358	0.000
(Sub-Sahara Spring) - Regional Breeding	1.781	0.112	319	15.907	0.000
(Sub-Sahara Spring) - NW Africa Breeding	1.596	0.249	319	6.411	0.000
(Sub-Sahara Spring) - (Sub-Sahara Breeding)	2.327	0.153	319	15.188	0.000
Local Breeding - Regional Breeding	0.021	0.135	319	0.155	1.000
Local Breeding - NW Africa Breeding	-0.163	0.260	319	-0.628	1.000
Local Breeding - (Sub-Sahara Breeding)	0.568	0.202	319	2.815	0.266
Regional Breeding - NW Africa Breeding	-0.184	0.235	319	-0.783	1.000
Regional Breeding - (Sub-Sahara Breeding)	0.547	0.170	319	3.208	0.102
NW Africa Breeding - (Sub-Sahara Breeding)	0.731	0.280	319	2.606	0.398

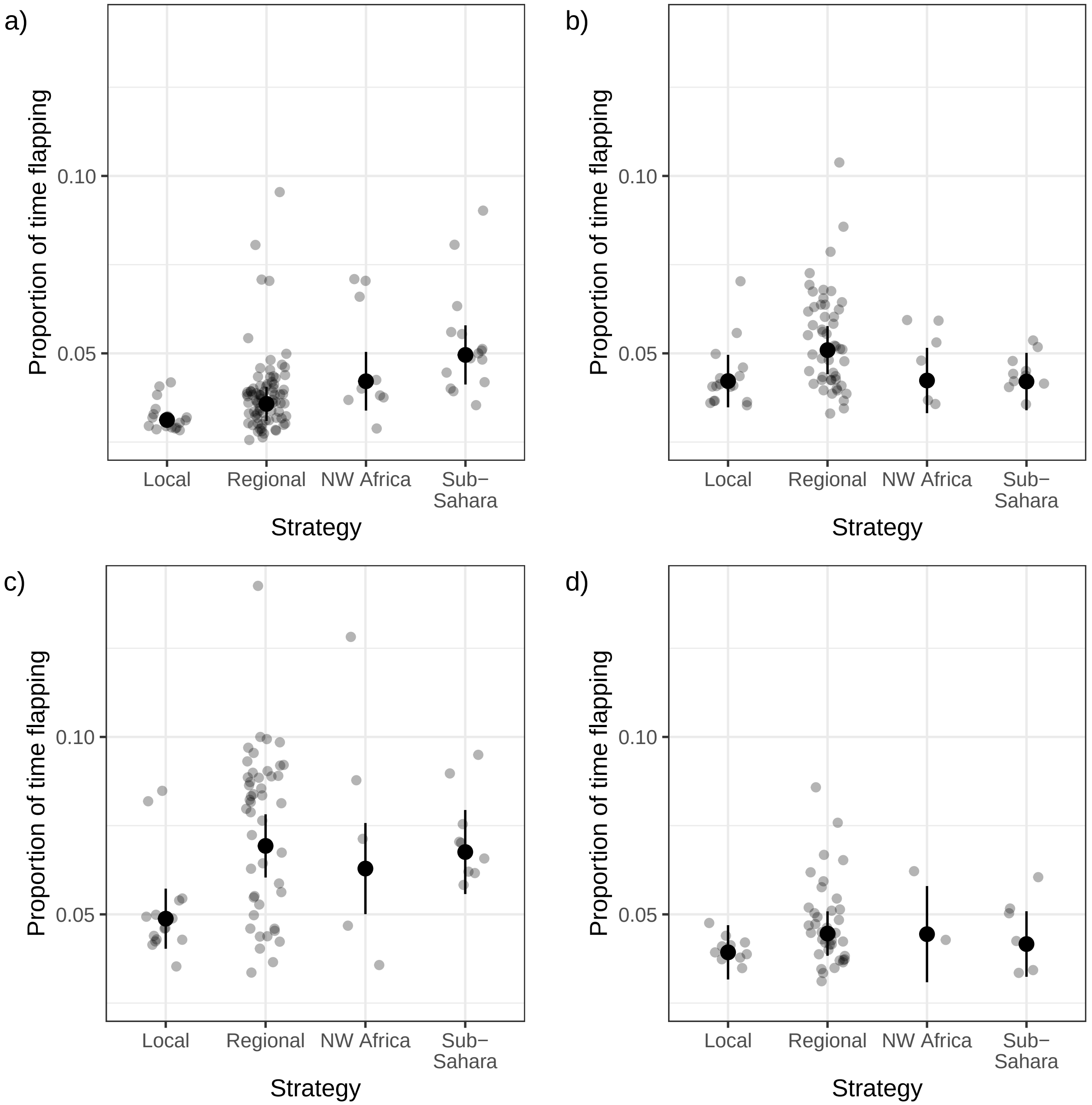

Fig S8: Relationship between the migratory strategy and flapping time, during the (a) autumn transition, (b) the wintering period, (c) the spring transition, and (d) the breeding period. Black dots are predicted estimates from the GLMM, vertical lines are the confidence intervals based on fixed-effect uncertainty, and grey dots are raw data.

Table S18: Tukey's contrasts multiple comparisons with adjusted p-values for the flapping time per migratory strategy and season.

contrast	estimate	SE	df	t.ratio	p.value
Local Autumn - Regional Autumn	-0.141	0.075	320	-1.868	0.888
Local Autumn - NW Africa Autumn	-0.311	0.107	320	-2.901	0.220
Local Autumn - (Sub-Sahara Autumn)	-0.481	0.096	320	-5.033	0.000
Local Autumn - Local Wintering	-0.312	0.079	320	-3.952	0.009
Local Autumn - Regional Wintering	-0.510	0.077	320	-6.610	0.000

Table S18 *(continued)*

contrast	estimate	SE	df	t.ratio	p.value
Local Autumn - NW Africa Wintering	-0.316	0.118	320	-2.678	0.350
Local Autumn - (Sub-Sahara Wintering)	-0.309	0.107	320	-2.897	0.222
Local Autumn - Local Spring	-0.465	0.078	320	-5.945	0.000
Local Autumn - Regional Spring	-0.837	0.076	320	-10.988	0.000
Local Autumn - NW Africa Spring	-0.734	0.114	320	-6.456	0.000
Local Autumn - (Sub-Sahara Spring)	-0.810	0.101	320	-8.057	0.000
Local Autumn - Local Breeding	-0.239	0.089	320	-2.674	0.352
Local Autumn - Regional Breeding	-0.370	0.079	320	-4.672	0.000
Local Autumn - NW Africa Breeding	-0.366	0.165	320	-2.226	0.678
Local Autumn - (Sub-Sahara Breeding)	-0.299	0.122	320	-2.455	0.508
Regional Autumn - NW Africa Autumn	-0.171	0.087	320	-1.967	0.841
Regional Autumn - (Sub-Sahara Autumn)	-0.341	0.073	320	-4.686	0.000
Regional Autumn - Local Wintering	-0.172	0.073	320	-2.358	0.580
Regional Autumn - Regional Wintering	-0.369	0.038	320	-9.806	0.000
Regional Autumn - NW Africa Wintering	-0.176	0.100	320	-1.766	0.926
Regional Autumn - (Sub-Sahara Wintering)	-0.169	0.087	320	-1.942	0.854
Regional Autumn - Local Spring	-0.324	0.073	320	-4.454	0.001
Regional Autumn - Regional Spring	-0.697	0.036	320	-19.385	0.000
Regional Autumn - NW Africa Spring	-0.593	0.094	320	-6.289	0.000
Regional Autumn - (Sub-Sahara Spring)	-0.670	0.079	320	-8.463	0.000
Regional Autumn - Local Breeding	-0.098	0.085	320	-1.151	0.999
Regional Autumn - Regional Breeding	-0.230	0.042	320	-5.507	0.000
Regional Autumn - NW Africa Breeding	-0.226	0.151	320	-1.493	0.983
Regional Autumn - (Sub-Sahara Breeding)	-0.158	0.105	320	-1.509	0.981
NW Africa Autumn - (Sub-Sahara Autumn)	-0.170	0.104	320	-1.626	0.963
NW Africa Autumn - Local Wintering	-0.001	0.106	320	-0.011	1.000
NW Africa Autumn - Regional Wintering	-0.198	0.088	320	-2.259	0.654
NW Africa Autumn - NW Africa Wintering	-0.005	0.109	320	-0.047	1.000
NW Africa Autumn - (Sub-Sahara Wintering)	0.002	0.115	320	0.017	1.000
NW Africa Autumn - Local Spring	-0.153	0.106	320	-1.453	0.987
NW Africa Autumn - Regional Spring	-0.526	0.087	320	-6.029	0.000
NW Africa Autumn - NW Africa Spring	-0.423	0.105	320	-4.018	0.007
NW Africa Autumn - (Sub-Sahara Spring)	-0.499	0.109	320	-4.579	0.001
NW Africa Autumn - Local Breeding	0.073	0.115	320	0.634	1.000
NW Africa Autumn - Regional Breeding	-0.059	0.090	320	-0.658	1.000
NW Africa Autumn - NW Africa Breeding	-0.055	0.160	320	-0.345	1.000
NW Africa Autumn - (Sub-Sahara Breeding)	0.012	0.128	320	0.097	1.000
(Sub-Sahara Autumn) - Local Wintering	0.169	0.094	320	1.800	0.915
(Sub-Sahara Autumn) - Regional Wintering	-0.028	0.074	320	-0.384	1.000
(Sub-Sahara Autumn) - NW Africa Wintering	0.165	0.116	320	1.424	0.989
(Sub-Sahara Autumn) - (Sub-Sahara Wintering)	0.172	0.085	320	2.022	0.811
(Sub-Sahara Autumn) - Local Spring	0.017	0.093	320	0.177	1.000
(Sub-Sahara Autumn) - Regional Spring	-0.356	0.073	320	-4.875	0.000
(Sub-Sahara Autumn) - NW Africa Spring	-0.253	0.111	320	-2.270	0.646
(Sub-Sahara Autumn) - (Sub-Sahara Spring)	-0.329	0.077	320	-4.253	0.003
(Sub-Sahara Autumn) - Local Breeding	0.243	0.104	320	2.341	0.593
(Sub-Sahara Autumn) - Regional Breeding	0.111	0.076	320	1.452	0.987
(Sub-Sahara Autumn) - NW Africa Breeding	0.115	0.163	320	0.704	1.000

Table S18 *(continued)*

contrast	estimate	SE	df	t.ratio	p.value
(Sub-Sahara Autumn) - (Sub-Sahara Breeding)	0.182	0.103	320	1.777	0.923
Local Wintering - Regional Wintering	-0.197	0.075	320	-2.633	0.379
Local Wintering - NW Africa Wintering	-0.004	0.117	320	-0.034	1.000
Local Wintering - (Sub-Sahara Wintering)	0.003	0.105	320	0.030	1.000
Local Wintering - Local Spring	-0.152	0.074	320	-2.046	0.797
Local Wintering - Regional Spring	-0.525	0.074	320	-7.100	0.000
Local Wintering - NW Africa Spring	-0.421	0.112	320	-3.756	0.019
Local Wintering - (Sub-Sahara Spring)	-0.498	0.099	320	-5.039	0.000
Local Wintering - Local Breeding	0.074	0.086	320	0.857	1.000
Local Wintering - Regional Breeding	-0.058	0.077	320	-0.750	1.000
Local Wintering - NW Africa Breeding	-0.054	0.164	320	-0.330	1.000
Local Wintering - (Sub-Sahara Breeding)	0.014	0.120	320	0.114	1.000
Regional Wintering - NW Africa Wintering	0.193	0.100	320	1.924	0.863
Regional Wintering - (Sub-Sahara Wintering)	0.200	0.088	320	2.277	0.641
Regional Wintering - Local Spring	0.045	0.075	320	0.602	1.000
Regional Wintering - Regional Spring	-0.328	0.037	320	-8.762	0.000
Regional Wintering - NW Africa Spring	-0.224	0.095	320	-2.358	0.581
Regional Wintering - (Sub-Sahara Spring)	-0.301	0.080	320	-3.745	0.019
Regional Wintering - Local Breeding	0.271	0.087	320	3.111	0.132
Regional Wintering - Regional Breeding	0.139	0.043	320	3.204	0.103
Regional Wintering - NW Africa Breeding	0.143	0.152	320	0.943	1.000
Regional Wintering - (Sub-Sahara Breeding)	0.211	0.106	320	1.992	0.828
NW Africa Wintering - (Sub-Sahara Wintering)	0.007	0.125	320	0.057	1.000
NW Africa Wintering - Local Spring	-0.148	0.117	320	-1.271	0.997
NW Africa Wintering - Regional Spring	-0.521	0.100	320	-5.212	0.000
NW Africa Wintering - NW Africa Spring	-0.417	0.115	320	-3.637	0.028
NW Africa Wintering - (Sub-Sahara Spring)	-0.494	0.120	320	-4.124	0.005
NW Africa Wintering - Local Breeding	0.078	0.125	320	0.623	1.000
NW Africa Wintering - Regional Breeding	-0.054	0.102	320	-0.528	1.000
NW Africa Wintering - NW Africa Breeding	-0.050	0.166	320	-0.301	1.000
NW Africa Wintering - (Sub-Sahara Breeding)	0.018	0.138	320	0.128	1.000
(Sub-Sahara Wintering) - Local Spring	-0.155	0.105	320	-1.481	0.984
(Sub-Sahara Wintering) - Regional Spring	-0.528	0.087	320	-6.052	0.000
(Sub-Sahara Wintering) - NW Africa Spring	-0.425	0.121	320	-3.510	0.042
(Sub-Sahara Wintering) - (Sub-Sahara Spring)	-0.501	0.088	320	-5.712	0.000
(Sub-Sahara Wintering) - Local Breeding	0.071	0.114	320	0.620	1.000
(Sub-Sahara Wintering) - Regional Breeding	-0.061	0.090	320	-0.677	1.000
(Sub-Sahara Wintering) - NW Africa Breeding	-0.057	0.170	320	-0.337	1.000
(Sub-Sahara Wintering) - (Sub-Sahara Breeding)	0.010	0.110	320	0.095	1.000
Local Spring - Regional Spring	-0.373	0.074	320	-5.045	0.000
Local Spring - NW Africa Spring	-0.269	0.112	320	-2.404	0.546
Local Spring - (Sub-Sahara Spring)	-0.346	0.099	320	-3.507	0.042
Local Spring - Local Breeding	0.226	0.085	320	2.649	0.369
Local Spring - Regional Breeding	0.094	0.077	320	1.225	0.998
Local Spring - NW Africa Breeding	0.098	0.164	320	0.600	1.000
Local Spring - (Sub-Sahara Breeding)	0.166	0.120	320	1.381	0.992
Regional Spring - NW Africa Spring	0.104	0.095	320	1.094	0.999
Regional Spring - (Sub-Sahara Spring)	0.027	0.080	320	0.340	1.000

Table S18 *(continued)*

contrast	estimate	SE	df	t.ratio	p.value
Regional Spring - Local Breeding	0.599	0.086	320	6.934	0.000
Regional Spring - Regional Breeding	0.467	0.042	320	11.172	0.000
Regional Spring - NW Africa Breeding	0.471	0.152	320	3.108	0.133
Regional Spring - (Sub-Sahara Breeding)	0.539	0.105	320	5.116	0.000
NW Africa Spring - (Sub-Sahara Spring)	-0.077	0.116	320	-0.662	1.000
NW Africa Spring - Local Breeding	0.495	0.121	320	4.107	0.005
NW Africa Spring - Regional Breeding	0.364	0.097	320	3.756	0.019
NW Africa Spring - NW Africa Breeding	0.367	0.161	320	2.276	0.641
NW Africa Spring - (Sub-Sahara Breeding)	0.435	0.134	320	3.241	0.093
(Sub-Sahara Spring) - Local Breeding	0.572	0.108	320	5.282	0.000
(Sub-Sahara Spring) - Regional Breeding	0.440	0.083	320	5.330	0.000
(Sub-Sahara Spring) - NW Africa Breeding	0.444	0.166	320	2.674	0.352
(Sub-Sahara Spring) - (Sub-Sahara Breeding)	0.512	0.105	320	4.871	0.000
Local Breeding - Regional Breeding	-0.132	0.089	320	-1.479	0.984
Local Breeding - NW Africa Breeding	-0.128	0.170	320	-0.754	1.000
Local Breeding - (Sub-Sahara Breeding)	-0.060	0.128	320	-0.470	1.000
Regional Breeding - NW Africa Breeding	0.004	0.153	320	0.025	1.000
Regional Breeding - (Sub-Sahara Breeding)	0.071	0.108	320	0.664	1.000
NW Africa Breeding - (Sub-Sahara Breeding)	0.068	0.180	320	0.377	1.000

Section S3: Multievent Model Design

Matrix representations with departure states in rows and arrival states in columns are commonly used in multievent models (See for instance Sanz-Aguilar et al., 2012). The initial state probabilities corresponded to the probability that a newly marked individual was an adult with an active GPS logger (Aa), an adult that has lost the GPS logger or its signal (Ai), a recently dead individual with an active GPS logger (Ra) and a long dead individual (LD). Here initial state probability (τ) was certainly known for every individual, as all individuals started as alive with an active GPS device deployed $(thus, \tau = 1)$.

$$IS = \begin{matrix} Aa & Ai & Ra & LD \\ \tau & 0 & 0 & 0 \end{matrix} \text{ Matrix 1}$$

We decomposed the transition between the state's probabilities into two steps: the first step corresponded to the probability of losing the GPS device or its signal (ψ, matrix 2) and the probability of survival (ϕ, matrix 3).

$$GPS\ loss = \begin{matrix} & Aa & Ai & Ra & LD \\ Aa & \sigma & 1-\sigma & 0 & 0 \\ Ai & 0 & 1 & 0 & 0 \\ Ra & 0 & 0 & 1 & 0 \\ LD & 0 & 0 & 0 & 1 \end{matrix} \text{ Matrix 2}$$

$$\text{Survival} = \begin{matrix} & Aa & Ai & Ra & LD \\ Aa & \Phi & 0 & 1-\Phi & 0 \\ Ai & 0 & \Phi & 0 & 1-\Phi \\ Ra & 0 & 0 & 0 & 1 \\ LD & 0 & 0 & 0 & 1 \end{matrix} \text{ Matrix 3}$$

The event probabilities (matrix 4) corresponded to the resighting probabilities (*p*) and the recovery probability (*r*). Since all recoveries were from individuals with active GPS loggers, we fixed recovery probability to one for all models (*r=1).*

$$\text{Survival} = \begin{matrix} & NS & SAa & SAi & SDa \\ Aa & 1-p & p & 0 & 0 \\ Ai & 1-p & 0 & p & 0 \\ Ra & 0 & 0 & 0 & r \\ LD & 1 & 0 & 0 & 0 \end{matrix} \text{ Matrix 4}$$
